# Nanotechnology as a Catalyst for Reintroducing Abandoned or Therapeutically Constrained Antimicrobial Agents: Opportunities and Translational Challenges

**DOI:** 10.1155/bmri/9486547

**Published:** 2026-11-08

**Authors:** Babatunde Ibrahim Olowu, Olalekan Toheeb Okewale, Abdulhakeem Opeyemi Azeez, Shammah Oluwaseyi Adeyemi

**Affiliations:** ^1^ Department of Veterinary Microbiology and Pathology College of Veterinary Medicine Washington State University Pullman Washington USA wsu.edu; ^2^ Department of Veterinary Pharmacology and Toxicology Faculty of Veterinary Medicine University of Ibadan Ibadan Nigeria ui.edu.ng; ^3^ Department of Veterinary Microbiology Faculty of Veterinary Medicine University of Ilorin Ilorin Nigeria unilorin.edu.ng; ^4^ Department of Veterinary Biochemistry Faculty of Veterinary Medicine University of Ibadan Ibadan Nigeria ui.edu.ng

**Keywords:** anti-infective therapeutics, drug delivery systems, drug repurposing, nanomedicine, nanotechnology, targeted drug delivery, translational therapeutics

## Abstract

The global rise in antimicrobial resistance, increasing rates of emerging and re‐emerging viral infections, and the persistent burden of fungal and parasitic diseases have intensified the need for alternative anti‐infective therapeutic strategies; a challenge further compounded by the diminishing pipeline of novel therapeutic agents. Historically, numerous compounds showed potent broad‐spectrum activity against bacterial, viral, fungal, and parasitic pathogens but failed to achieve widespread clinical adoption due to significant limitations, including systemic toxicity, poor selectivity, rapid degradation, unfavorable pharmacokinetics, and off‐target host damage. Consequently, many of these promising therapeutic agents were abandoned despite their demonstrated efficacy. Advances in nanotechnology have revitalized interest in these historically neglected compounds by enabling targeted delivery, controlled release, improved biodistribution, and reduced toxicity, offering strategies to improve the therapeutic index of agents once considered clinically impractical. Specifically, this review examines how nanotechnology can reshape the translational potential of therapeutically constrained anti‐infective agents and highlights key examples, including halogen‐based antimicrobials, metal‐derived therapeutics, reactive oxygen and nitrogen species, and membrane‐active compounds. We discuss how these technologies address historical barriers to therapeutic application and critically evaluate the remaining barriers hindering clinical translation and long‐term biological interactions. Collectively, we propose that the next generation of anti‐infective therapeutics may emerge not solely from discovering entirely new compounds, but from strategically reengineering abandoned agents using advanced nanotechnology‐enabled delivery systems.

## 1. Introduction

Infectious diseases remain a major global health challenge despite substantial advances in antimicrobial therapy, vaccination, and disease prevention. Antimicrobial resistance (AMR), emerging and re‐emerging viral infections, persistent fungal diseases, and neglected tropical and parasitic diseases expose important limitations in the current therapeutic armamentarium [1–3]. Resistant pathogens increasingly compromise established therapies and narrow treatment options, whereas the development of new anti‐infective agents remains scientifically and economically challenging [1–6]. These limitations have encouraged renewed interest not only in new molecular entities, but also in older, forgotten, or previously underused anti‐infective compounds [7–10]. Historically, several such agents retained useful antimicrobial activity but were discontinued, displaced, restricted to limited indications, or failed to progress because of toxicity, poor selectivity, instability, unfavorable pharmacokinetics, or narrow therapeutic windows [11–16].

This history raises an important question: Can advances in formulation and delivery justify reconsidering agents previously regarded as unsuitable for systemic or broader therapeutic use? For some compounds, limited clinical utility reflects difficulties in controlling concentration, distribution, stability, or exposure rather than insufficient antimicrobial activity [17, 18]. Poor pharmacokinetics can produce inadequate exposure at the infection site while increasing exposure in nontarget tissues [18, 19]. Similarly, nonspecific membrane activity or reactive chemical mechanisms may be effective against pathogens but are difficult to administer safely because the same mechanisms can damage both pathogens and host cells, limiting safe use [11, 20, 21]. However, “abandoned” encompasses different therapeutic histories: Some compounds were discontinued, whereas others remained available as topical agents, second‐line therapies, or treatments for narrowly defined indications [22–24]. Their reconsideration therefore requires identifying the specific barrier that originally limited their use.

The revival of older antibiotics has been examined extensively in the context of AMR [9, 22, 23, 25, 26]. A broader, directly overlapping body of reviews has also examined nanotechnology‐based antimicrobial delivery, including protection from degradation, controlled release, altered biodistribution, intracellular delivery, and targeting of infected tissues or microbial communities [17, 18, 27–29]. Other reviews address targeted nanomaterials, hybrid nanocarriers, infection‐responsive systems, and codelivery strategies for improving antimicrobial efficacy or overcoming resistance [30–32]. These mechanisms provide the foundation for the present review. Its distinct contribution is to organize and evaluate representative cases according to the historical barrier that constrained each agent′s therapeutic use, rather than primarily by nanoparticle platform or resistance mechanism. This framework links the original limitation to the proposed nanoengineering intervention, and the evidence needed to show that the limitation has been meaningfully addressed. It thereby distinguishes general improvements in antimicrobial delivery from evidence supporting therapeutic reconsideration.

Advances in nanomedicine provide a technological basis for such reassessment. Nanoparticle‐based systems can improve solubility, protect unstable compounds, regulate release, modify tissue localization, and potentially reduce exposure of healthy tissues [18, 33–35]. Lipid‐based carriers, polymeric nanoparticles, metallic nanostructures, nanogels, peptide‐based hydrogels, and stimuli‐responsive systems have therefore expanded the range of strategies available for improving therapeutic delivery [17, 36]. Surface modification can further influence circulation, cellular uptake, tissue penetration, and interaction with biological barriers [37]. Nanoenabled formulations have consequently been investigated for compounds whose earlier use was constrained by toxicity or unfavorable pharmacological properties [11, 38–43].

Such reformulation could complement de novo anti‐infective discovery, but improved delivery does not necessarily overcome intrinsic toxicity, and enhanced in vitro activity does not establish clinical relevance [44, 45]. Nanomedicines may introduce additional challenges involving biodistribution, immunogenicity, persistence, and carrier‐associated toxicity [44, 46–48]. Clinical translation also requires reproducible manufacturing, batch consistency, characterization of critical quality attributes, scalable production, and regulatory compliance [46, 48–51]. The central question is therefore whether a nanoenabled formulation demonstrably addresses the barrier that previously restricted an agent′s use while maintaining an acceptable safety profile.

Against this background, this perspective examines nanotechnology′s potential to support the therapeutic reconsideration of abandoned or constrained anti‐infective agents. This perspective considers representative halogen‐based, metal‐based, reactive species‐based, and membrane‐active agents across bacterial, viral, fungal, and, where evidence supports inclusion, parasitic pathogens. Each case is assessed in relation to its historical limitation, the proposed nanoenabled solution, the supporting evidence, and the remaining barriers to clinical translation. This approach provides a basis for judging when reengineering an established agent may offer a credible route to renewed or expanded therapeutic use.

## 2. Previously Abandoned Antimicrobial Agents: Causes of Therapeutic Failure and Clinical Displacement

Many antimicrobial agents declined in clinical use not because they had lost intrinsic activity against their target pathogens, but because toxicity, pharmacological limitations, resistance, or the availability of safer and more convenient alternatives reduced their therapeutic value (Table 1). Colistin, polymyxin B, streptomycin, vancomycin, chloramphenicol, fosfomycin, and nitrofurantoin largely retained potent in vitro activity throughout the decades in which they fell out of favor, and several of them were eventually reintroduced once resistant organisms left clinicians with few remaining options ([2, 7, 12]; Table 1). The same pattern recurs well beyond the classical antimicrobials: Nourseothricin and hygromycin A were shelved decades ago for toxicity and narrow commercial appeal, respectively, only to be rediscovered against multidrug‐resistant Gram‐negatives and the Lyme disease spirochaete [84, 85], whereas the antileishmanial tartar emetic and the antiamoebic clioquinol were withdrawn for cardiotoxicity and neurotoxicity, respectively, and are now being explored as nanocarrier formulations rather than reintroduced as free drug [39, 92]. The same molecules unusable in one era and indispensable in the next is itself instructive: It shows that abandonment, in most of these cases, was never really a verdict on their potency, but on challenges and limitations that surfaced once these drugs were used at scale; mechanisms of action that failed to distinguish sharply enough between microbial and host targets; pharmacokinetic behavior that made effective dosing difficult to predict; chemical structures prone to degradation; safety margins narrow enough that therapeutic and toxic exposures overlapped, compounded by biological phenomena such as heteroresistance and the inoculum effect; and a clinical, regulatory, and commercial environment that offered little incentive to resolve any of the above once alternatives appeared ([4, 8, 14, 25, 103–105]; Table 1).

**Table 1:** Previously abandoned antimicrobial agents: historical role, cause of decline, current status, and nanotechnology‐relevant design requirements.

Agent (class)	Historical role	Reason for decline/restriction	Dominant limitation	Current status	Nanodesign requirement	Key references
Colistin (polymyxin E) – lipopeptide	Isolated from *Paenibacillus polymyxa* subsp. *colistinus*; used systemically from the 1950s for Gram‐negative infection, then sidelined once aminoglycosides and fluoroquinolones offered comparable activity with less reported toxicity.	Nephrotoxicity reported in up to 60% and neurotoxicity in up to 27% of early parenteral cohorts, compounded by unpredictable CMS‐to‐colistin hydrolysis and inconsistent mg/IU dosing conventions across manufacturers.	Coupled membrane‐selectivity toxicity and prodrug pharmacokinetic instability.	Reintroduced from the 2000s as the last‐resort therapy for carbapenem‐resistant Enterobacterales, *A. baumannii*, and *P. aeruginosa*; formal international PK/PD dosing consensus only published in 2019, 70 years after its discovery in 1949.	Renal‐tubule‐sparing, site‐restricted carriers (liposomal/polymeric) limiting free‐colistin exposure to proximal tubule cells while preserving outer‐membrane activity; controlled release to bypass CMS hydrolysis variability.	[12]; [52]; [53]; [54]; [55]; [56]; [57]; [58]; [10]
Polymyxin B – lipopeptide	Isolated alongside colistin; administered as the active compound rather than a prodrug, avoiding conversion delay but sharing colistin′s toxicity profile.	Nephrotoxicity/neurotoxicity at systemic doses; poor lung penetration limits efficacy in pneumonia at tolerable IV doses.	Membrane selectivity combined with poor pulmonary biodistribution.	Revived for MDR Gram‐negative bacteraemia; increasingly combined with nebulized administration for nosocomial/ventilator‐associated pneumonia where IV dosing alone underdelivers to lung tissue.	Inhalable, lung‐targeted nanocarriers to raise local alveolar concentration without raising systemic renal exposure.	[59]; [60]; [61]; [12]; [62]
Vancomycin – glycopeptide	Isolated from *Streptomyces orientalis*; introduced as the first glycopeptide but rapidly marginalized once methicillin arrived in 1960, with impure early batches (Mississippi mud) driving disproportionate toxicity reports.	Poor manufacturing purity in early preparations, later compounded by a genuinely narrow AUC/MIC therapeutic index once purified formulations reached wide use.	Narrow therapeutic index requiring individualized, monitored dosing.	First‐line for MRSA since the 1980s once glycopeptide resistance forced reconsideration; dosing practice shifted from trough‐based to AUC‐guided monitoring only after 2020 consensus guidelines.	Controlled/sustained‐release carriers to flatten peak‐trough swings; silver‐nanoparticle coformulation explored to restore activity against reduced‐susceptibility strains.	[63]; [64]; [65]
Streptomycin – aminoglycoside	First antibiotic active against *Mycobacterium tuberculosis* (Waksman); cornerstone of early combination TB therapy before rapid monotherapy resistance emerged.	Off‐target binding to the structurally related mitochondrial 12S rRNA causes ototoxicity/nephrotoxicity; poor blood–brain barrier penetration historically required intrathecal dosing for TB meningitis at neurotoxic doses.	Off‐target mitochondrial ribosome binding plus CNS biodistribution failure.	Retained as a second‐line injectable for multidrug‐resistant tuberculosis where newer agents are unavailable or contraindicated.	Cochlear‐ and renal‐sparing targeted delivery (e.g., localized inner‐ear or renal‐avoidance carrier systems).	[66]; [26]; [19]
Chloramphenicol – amphenicol	Isolated from *Streptomyces venezuelae*; the first broad‐spectrum antibiotic, used against typhoid, bacterial meningitis, and rickettsial disease from the late 1940s.	Dose‐dependent, reversible bone‐marrow suppression in a substantial minority of patients, and a rare (~1 in 20,000–40,000) but frequently fatal, dose‐independent aplastic anemia, both traced to inhibition of mitochondrial ribosomal translation.	Mitochondrial off‐target toxicity in hematopoietic progenitor cells.	Withdrawn from routine systemic use in high‐income countries; retained topically/ophthalmically and, on cost grounds, still used systemically in some LMICs.	Marrow‐sparing encapsulation—silver‐nanoparticle‐conjugated, PCL–pluronic, nanosilica, or metal‐oxide‐doped carriers—to concentrate activity at the infection site and reduce systemic marrow exposure.	[67]; [68]; [69]; [70]; [71]
Nitrofurantoin – nitrofuran	Introduced as an oral agent for uncomplicated urinary tract infection; a multitarget free‐radical mechanism has kept resistance rates low since its introduction in 1953.	Active metabolites accumulate once glomerular filtration declines, producing pulmonary/hepatic toxicity chiefly in older patients; shortened treatment courses have also been linked to higher clinical failure rates.	Narrow safety margin specifically in reduced renal function.	Still first‐line, guideline‐recommended therapy for uncomplicated cystitis.	Renal function‐independent, urinary tract‐targeted delivery maintaining local bladder‐wall concentration without systemic metabolite accumulation.	[72]; [73]; [74]; [75]; [76]
Fosfomycin – phosphonic acid	Discovered from *Streptomyces fradiae*; a phosphoenolpyruvate‐mimicking mechanism gives it low cross‐resistance with other antibiotic classes.	The original calcium salt achieves only ~12% oral bioavailability, confining the drug to niche use until the tromethamine salt (34%–58% bioavailability) enabled single‐dose oral therapy; heteroresistance has since been documented in MDR *E. coli*.	Salt form‐dependent oral bioavailability.	Reserve oral agent for uncomplicated and, increasingly, complicated MDR urinary tract infection; IV disodium formulation used for systemic MDR infection.	Bioavailability‐enhancing oral nanocarriers (lipid‐ or polymer‐based) to raise and stabilize absorption independent of salt form.	[25]; [77]; [78]; [79]
Amphotericin B – polyene	Isolated from *Streptomyces nodosus*; introduced clinically in the late 1950s and remained the principal broad‐spectrum systemic antifungal for several decades (gold standard) despite severe infusion‐related toxicity.	Residual affinity for mammalian cholesterol alongside its target, fungal ergosterol, forms pores in renal tubular membranes at clinically active doses.	Narrow selectivity margin between fungal and host membrane sterols.	Retained as first‐line systemic therapy for invasive fungal infection, but almost exclusively in lipid‐based/liposomal formulations (e.g., AmBisome) that have already substantially reduced, though not eliminated, nephrotoxicity since the 1990s.	Further refinement of lipid‐based reformulation—the clearest existing proof‐of‐concept that nanocarrier encapsulation can rescue an abandoned‐by‐toxicity agent, and a template for other agents in this table.	[80]; [81]; [82]; [31]
Penicillin G – *β*‐lactam	Fleming′s 1928 discovery, mass‐produced from 1942 to 1943; the founding molecule of the antibiotic era.	Rapid hydrolysis by gastric acid prevented reliable oral absorption, confining early use to IM/IV administration; true penicillin allergy in a minority of patients further restricted use.	Acid lability under gastric conditions.	Superseded for oral use by acid‐stable congeners (penicillin V, aminopenicillins); the original formulation is retained for IV/IM use in syphilis, streptococcal, and other infections where its narrow spectrum is an advantage.	Enteric or acid‐protective encapsulation to enable oral bioavailability of the original, narrow‐spectrum molecule.	[83]
Nourseothricin (streptothricin‐F) – streptothricin	Isolated by Waksman and Woodruff; the first identified antibiotic with potent Gram‐negative activity, generating major early interest.	A small, unpublished 1940s Merck human trial found apparent kidney damage; development was abandoned and the underlying data never released, leaving the compound essentially untested until its re‐evaluation in 2023, approximately eight decades after its 1942 discovery.	Off‐target renal toxicity attributable to the unpurified congener mixture, not necessarily to streptothricin‐F itself.	Revived in 2023 after cryo‐EM structural work and toxicity reprofiling showed streptothricin‐F, purified to homogeneity, retains potent bactericidal activity against carbapenem‐resistant Enterobacterales and *A. baumannii* with markedly lower nephrotoxicity than the D congener or the crude mixture.	Congener‐selective, purified‐component delivery to prevent reintroduction of the more toxic streptothricin‐D component during manufacture or degradation.	[84]
Hygromycin A – aminocyclitol	Isolated from *Streptomyces hygroscopicus* (Eli Lilly); briefly evaluated for swine dysentery caused by *Brachyspira hyodysenteriae*.	Weak activity against most Gram‐positive and Gram‐negative bacteria made it commercially unviable outside that one veterinary niche; not withdrawn for toxicity.	Commercial/economic neglect rather than a safety or delivery failure.	Rediscovered in 2021 as a highly selective agent against spirochaetes, including *Borrelia burgdorferi* (Lyme disease) and *Treponema pallidum* (syphilis), with minimal effect on commensal gut flora.	Gut‐microbiome‐sparing oral delivery that exploits the drug′s intrinsic narrow spectrum rather than working around toxicity.	[85]
Fusidic acid – fusidane	A steroid‐like translation inhibitor from *Fusidium coccineum*, in continuous use across Europe, Australia, and Asia since 1962 for staphylococcal, including MRSA, infection.	Resistance emerges rapidly during monotherapy (frequency ~10^−6^–10^−8^ at 4–8× MIC), making combination use mandatory; separately, never successfully approved by the US FDA, reportedly for commercial/regulatory rather than scientific reasons.	Rapid monotherapy resistance combined with a persistent US regulatory gap.	Widely used outside the United States; in active US Phase III development (as CEM‐102/sodium fusidate) for chronic staphylococcal bone and joint infection.	Combination‐loaded or resistance‐suppressing carriers that codeliver a second agent to prevent monotherapy‐driven resistance selection.	[86]; [87]
Octapeptins – lipopeptide	A family of cyclic lipopeptides structurally related to the polymyxins, first reported in the 1970s.	At the time, activity against polymyxin‐resistant bacteria was treated as a laboratory curiosity with no commercial rationale, since polymyxin resistance was not yet a clinical problem.	Economic neglect predating the resistance crisis, not toxicity—octapeptins carry at least one fewer cationic charge than polymyxins and a superior preclinical renal safety profile.	Actively being redeveloped (first total chemical syntheses only recently reported) as polymyxin alternatives active against colistin‐resistant *Acinetobacter*, *Pseudomonas*, and *Klebsiella*.	Renal‐sparing lipopeptide carriers building on octapeptins′ inherently lower nephrotoxicity relative to polymyxins.	[88]
Gramicidin – peptide ionophore	Isolated by Dubos; the first antibiotic ever purified and commercialized for topical use, predating even penicillin′s clinical introduction.	Its ion‐channel‐forming mechanism causes potassium efflux and rapid hemolysis at systemic concentrations, so systemic use was never clinically viable.	Nonselective, mechanism‐intrinsic membrane toxicity common to mammalian and bacterial cells alike.	Confined to topical/ophthalmic formulations (e.g., triple‐antibiotic ointment) since its isolation in 1939; recent machine‐learning‐guided analogue redesign has achieved a 42‐fold expansion of the in vitro therapeutic window.	Site‐restricted topical/wound nanocarriers physically preventing systemic absorption while concentrating activity at superficial infection sites.	[16]; [89]
Ketoconazole (oral) – imidazole	The first broad‐spectrum oral antifungal, used for decades as first‐line systemic/superficial mycosis therapy before triazoles became available.	Hepatotoxicity evidence emerged within its first years of use; a comprehensive FDA/EMA risk‐benefit review found hepatotoxicity rates of 3.6%–4.3% with no clear dose‐relationship, alongside adrenal insufficiency from off‐target steroidogenesis inhibition.	Off‐target inhibition of cytochrome P450 enzymes and adrenal steroidogenesis.	Oral formulation withdrawn in the EU and Australia in 2013; US FDA restricted labeling to endemic mycoses only, though off‐label prescribing for skin/nail infection has continued. Topical formulations remain unrestricted (minimal systemic absorption).	Liver‐sparing, targeted antifungal carriers that would allow systemic redeployment without hepatic/adrenal exposure.	[13]
Clioquinol – halogenated 8‐hydroxyquinoline	Iodochlorhydroxyquin, used from the 1950s to 1970 as an oral antiamoebic/antidiarrhoeal, especially widely in Japan.	Linked to roughly 10,000 cases of subacute myelo‐optic neuropathy (SMON) in Japan between 1955 and 1970—sensory disturbance, weakness, and visual loss; the association remains scientifically debated but was sufficient to trigger a 1970 Japanese ban and earlier US restriction to topical use.	Off‐target neurotoxicity; ~40% of surviving SMON patients still unable to walk independently 32 years after the ban.	Oral/systemic use banned since 1970–71; retained in topical antifungal/antiseptic formulations; being actively reformulated as nanocrystals and polymeric nanocarriers for dermatomycosis, deliberately avoiding systemic exposure.	Nanocrystal or nanocarrier formulation restricted to topical/dermatological application, exploiting improved local bioavailability without systemic absorption.	[90]; [91]; [92]; [93]
Tartar emetic (antimony potassium tartrate) – trivalent antimonial	First used successfully against cutaneous leishmaniasis in 1912 and visceral leishmaniasis in 1915; later extended to schistosomiasis through the mid‐20th century.	Dose‐dependent cardiotoxicity—ECG changes (T‐wave flattening/inversion in up to two‐thirds of patients, occasional acute vascular collapse)—alongside severe gastrointestinal intolerance.	Narrow therapeutic index between antiparasitic and cardiotoxic exposure.	Superseded by praziquantel for schistosomiasis (1970s) and pentavalent antimonials for leishmaniasis; being revived experimentally as a liposomal formulation that improves the pharmacokinetic profile and mitigates cardiotoxicity in murine models.	Cardioprotective liposomal/PEGylated encapsulation restricting antimony exposure to phagocytic/reticuloendothelial tissue and away from cardiac tissue.	[15]; [39]; [94]; [95]
Quinacrine (mepacrine) – acridine	One of the first synthetic antimalarials, developed in the 1920s–30s and used extensively by Allied forces in WWII.	Neuropsychiatric toxic psychosis (confusion, hallucinations, mania‐like presentations) at therapeutic doses, alongside reversible yellow skin discoloration; displaced once chloroquine offered comparable efficacy with substantially better tolerability.	Off‐target central nervous system toxicity.	No FDA‐approved formulation marketed in the United States since around 2020, though available via compounding pharmacies; retained off‐label for cutaneous/discoid lupus erythematosus.	Nanotechnology improvement research on oncology and neurodegeneration.	[96]
Silver (colloidal/ionic) – antiseptic metal	Standard systemic and topical antisepsis—wound infection, general sepsis, and (as silver arsphenamine) syphilis—into the early 20th century, alongside Credé′s 1881 silver‐nitrate ocular prophylaxis.	Physician‐directed use declined rapidly once sulfonamides (1930s) and penicillin (1940s) offered comparable efficacy without argyria, the irreversible blue‐gray skin discolouration produced by cumulative silver exposure.	Off‐target toxicity (argyria) compounded by clinical displacement once true antibiotics arrived.	Confined to narrow topical/prophylactic niches—silver sulfadiazine for burns since the 1960s, impregnated wound dressings—before renewed interest driven by rising antibiotic resistance.	Nanoscale AgNP formulations achieve deeper wound‐bed penetration and lower effective dose than bulk/ionic silver, restoring biofilm activity while keeping cumulative exposure below the argyria threshold.	[97]; [98]; [65]; [99]
Iodine (free/tincture) – halogen antiseptic	Tincture of iodine and Lugol′s solution were the dominant chemical antiseptics for wound and skin disinfection from the 19th century through the mid‐20th.	Uncomplexed free iodine (I2/triiodide) causes chemical burns and deep skin staining, and liberal or prolonged application produces systemic iodine toxicity (thyrotoxicosis, hypothyroidism); contemporary formulators explicitly attributed its declining favor to “relative instability and short period of activity” as the ethanol vehicle evaporates and free iodine is consumed.	Coupled off‐target toxicity and chemical instability (uncontrolled, short‐lived release).	Superseded for most clinical antisepsis by iodophors (povidone‐iodine) from the 1950s, which reduce free‐I2 activity 30‐ to 50‐fold via macromolecular complexation; free tincture retained only for niche pre‐surgical thyroid use.	Nanocarrier‐controlled iodine release (chitosan, MOF, photothermal‐triggered systems) extends the same “tame the free halogen” logic iodophors pioneered, but with spatial and temporal control iodophors cannot achieve.	[100]; [101]
Bismuth (subgallate/subnitrate) – antisyphilitic heavy metal	First‐line antisyphilitic (alongside mercurials and arsenicals) before penicillin, and used more broadly for GI and wound infection.	A neurotoxicity (encephalopathy) epidemic linked to prolonged bismuth subgallate/subnitrate use was reported in France in the 1970s; separately, bismuth was entirely superseded as an antimicrobial once penicillin proved dramatically more effective against syphilis from the 1940s.	Off‐target neurotoxicity (specific salts) combined with clinical displacement by penicillin.	Systemic antimicrobial bismuth is essentially extinct; bismuth subsalicylate/subcitrate persist only for GI indications (*H. pylori* triple therapy, travelers′ diarrhea) at doses with minimal systemic absorption.	Bismuth nanoparticle and BisBAL (bismuth‐dimercaprol) systems are being explored for antibiofilm activity, deliberately exploiting bismuth′s low intestinal‐absorption profile to limit the systemic exposure that caused 1970s‐era toxicity.	[102]

Abbreviations: AUC/MIC, area under the concentration–time curve to minimum inhibitory concentration ratio; CMS, colistin methanesulfonate; MDR, multidrug‐resistant; PK/PD, pharmacokinetic/pharmacodynamic.

### 2.1. Poor Selectivity, Host Toxicity, and Off‐Target Damage

Many old agents emerged from phenotypic screens for antimicrobial potency; selectivity against host cells was rarely a design criterion. Where the microbial target had a close structural or evolutionary counterpart in human cells, this omission proved costly. Aminoglycosides bind the bacterial 30S ribosomal 16S rRNA and the structurally related mitochondrial 12S rRNA, and the resulting disruption of mitochondrial translation in cochlear and renal tubular cells underlies the ototoxicity and nephrotoxicity that have curtailed their use [26, 66]. Chloramphenicol follows the same logic in bone marrow, producing dose‐dependent suppression and, rarely, fatal aplastic anemia [67]. Trovafloxacin illustrates a mechanistically distinct problem: This fluoroquinolone suppressed hepatic mitochondrial function idiosyncratically, in a small and unpredictable fraction of patients, causing acute liver failure that could not be foreseen from trial data and forcing its withdrawal from Europe and near‐total restriction in the United States within 2 years of launch [106, 107].

Membrane‐active agents illustrate a related but distinct problem: Rather than a single conserved target, they exploit physicochemical differences between microbial and mammalian membranes that are rarely absolute. Colistin and polymyxin B permeabilize Gram‐negative membranes via cationic, amphipathic interactions that also damage renal tubular and neuronal membranes, producing the nephro‐ and neurotoxicity that drove their near‐total withdrawal by the early 1980s [12, 55, 61, 108, 109]. Amphotericin B shows the same pattern in reverse, binding fungal ergosterol preferentially over mammalian cholesterol yet still forming pores in cholesterol‐containing renal membranes at clinical doses [80]. Gramicidin is the most extreme case: Its ion‐channel‐forming mechanism is so mammalian‐cell‐agnostic that systemic use has never proven viable, and 87 years after its isolation by Dubos in 1939, it remains confined to topical formulations [16, 89].

These toxicity signals do not all share the same origin—a distinction that matters for identifying which agents are genuinely suited to nanocarrier‐based rescue. Early vancomycin′s poor manufacturing purity, rather than the molecule itself, drove much of its reported ototoxicity before purification methods improved [63]: an artifact of production rather than an intrinsic pharmacological liability. By contrast, the toxicities of several other agents withdrawn appear intrinsic to the molecule itself. Clioquinol, an iodinated antiamoebic used for two decades, was withdrawn after being linked to roughly 10,000 cases of subacute myelo‐optic neuropathy in Japan [90]. Oral ketoconazole, the first broad‐spectrum oral antifungal, was similarly restricted in the European Union, Australia, and the United States once idiosyncratic hepatotoxicity and adrenal suppression were judged to outweigh its benefit in routine infections [13]. Quinacrine, the first widely used synthetic antimalarial, was displaced by chloroquine largely because of toxic psychosis at therapeutic doses [96]. Two heavy‐metal antimicrobials that predate the antibiotic era altogether show the same logic in a different tissue: cumulative exposure to systemic silver produces argyria, an irreversible blue‐gray skin discoloration [97], whereas prolonged use of specific bismuth salts (subgallate, subnitrate) was linked to a documented neurotoxicity epidemic in France in the 1970s. Across all these cases, it is the clinical consequence, not its molecular origin, that selective nanocarrier delivery is designed to address.

### 2.2. Unfavorable Pharmacokinetics, Bioavailability, and Biodistribution

A second recurring failure mode concerns achieving an adequate, predictable concentration of the active drug at the infection site rather than the drug′s intrinsic activity. Colistin is dosed intravenously as the inactive prodrug colistin methanesulfonate (CMS), which hydrolyses slowly and incompletely in plasma; tissue penetration is limited by the molecule′s large, cationic structure, and interpatient variability in conversion was managed empirically for decades before population pharmacokinetic modeling became standard [25, 52, 56, 110]. Polymyxin B avoids the prodrug delay but still requires supplementary inhaled therapy to reach effective lung concentrations in nosocomial pneumonia [59, 60].

Fosfomycin shows how much a single formulation choice can swing bioavailability: Its calcium salt achieves roughly 12% oral bioavailability, whereas the tromethamine salt reaches 34%–58%, a difference that determined which formulation became clinically viable [25, 77]. The intravenous disodium formulation, developed to reach infections oral dosing cannot, trades this problem for a different one: Fosfomycin inhibits MurA, the bacterial enolpyruvyl transferase that catalyzes the first committed step of peptidoglycan synthesis [78], and because the drug is highly hydrophilic and cleared almost entirely by glomerular filtration, plasma exposure varies sharply with renal function—a particular problem in intensive care, where acute kidney injury is common. Each gram of fosfomycin disodium also delivers roughly 14 mEq of sodium, so a standard 16‐g/day regimen for severe infection can approach 230 mEq of sodium daily, a clinically significant load in patients with cardiac or renal comorbidity [111]. Historically, oral absorption problems were not confined to peptides: Penicillin G is hydrolysed by gastric acid, restricting early use to parenteral administration until acid‐stable congeners were developed [83], and streptomycin barely crosses the blood–brain barrier, requiring intrathecal administration for tuberculous meningitis at doses associated with substantial neurotoxicity [19].

A related, underappreciated biodistribution barrier is the biofilm itself: Chronic *Pseudomonas aeruginosa* infection in cystic fibrosis airways proceeds under anaerobic, quorum‐sensing‐governed conditions that many of these agents were never designed to penetrate, a mismatch between drug and microenvironment that recurs across chronic and device‐associated infections regardless of antibiotic class [112]. In each case, the anti‐infective identified as active in vitro and the drug clinicians could deliver were, functionally, different molecules, precisely the gap that nanocarrier‐based delivery targets.

### 2.3. Chemical Instability, Rapid Degradation, and Loss of Bioactivity

Chemical instability can substantially limit antimicrobial efficacy by reducing the amount of active compound available during storage, administration, or systemic exposure. Hydrolysis, oxidation, deamidation, proteolytic degradation, and other physicochemical changes can alter antimicrobial structure and activity, resulting in unpredictable exposure or premature loss of bioactivity [11, 56]. CMS illustrates a related but distinct problem. As an inactive prodrug, CMS undergoes spontaneous hydrolysis to generate active colistin, with the rate of conversion strongly influenced by temperature and biological medium [53, 54]. Conversion occurs alongside renal elimination of CMS, contributing to considerable interpatient variability in exposure to active colistin [52, 55, 110]. Historically, differences in how colistin products expressed dosing units further complicated prediction and comparison of active drug exposure [52, 58]. Thus, for prodrugs such as CMS, the problem is not degradation alone but the inability to precisely control when and where the pharmacologically active species is generated.

Other antimicrobial classes are limited by direct degradation or loss of the chemical structure required for activity. Peptide‐based antimicrobials are susceptible to enzymatic and physicochemical degradation, which contributes to short biological persistence and limits systemic application despite their potent antimicrobial activity [11, 56, 113]. Similarly, the *β*‐lactam ring illustrates the same principle at the level of chemical structure: It is intrinsically susceptible to hydrolysis, which is the underlying degradation chemistry behind penicillin G′s gastric‐acid instability [83]. Halogen‐based agents present another formulation challenge. Free iodine is highly reactive and has limited persistence, whereas direct exposure at effective concentrations can cause local irritation and damage to host tissue [101, 114]. The development of iodophors represented an early strategy to regulate free iodine availability, and subsequent polymeric and nanoscale systems have extended this principle toward more controlled stabilization and release [38, 40, 101].

Importantly, instability, pharmacokinetics, and toxicity are often interconnected rather than independent barriers. Premature degradation may reduce antimicrobial exposure at the infection site, whereas uncontrolled conversion or release can increase exposure of healthy tissues to active or reactive species. Modern delivery systems offer a means of addressing this relationship by protecting labile agents during delivery and controlling their release or activation at the intended site [17, 35, 115].

### 2.4. Narrow Therapeutic Index and Dose‐Limiting Constraints

For several antimicrobial agents, clinical utility has been constrained by a narrow therapeutic window in which the exposure required for effective pathogen control approaches that associated with host toxicity. This problem is particularly evident for agents whose antimicrobial mechanisms are insufficiently selective or whose systemic distribution exposes susceptible host tissues to pharmacologically active concentrations. Vancomycin exemplifies this challenge: Inadequate exposure can compromise treatment of serious methicillin‐resistant *Staphylococcus aureus* (MRSA) infections, whereas excessive exposure increases the risk of nephrotoxicity. Contemporary guidelines therefore recommend an area under the concentration–time curve to minimum inhibitory concentration ratio (AUC/MIC) of 400–600 mg·h/L for serious MRSA infections, replacing trough concentration alone as the preferred monitoring approach because higher trough‐based exposures were associated with increased nephrotoxicity [63, 64]. Aminoglycosides face a comparable dose‐limiting problem, with nephrotoxicity and irreversible ototoxicity necessitating careful dose adjustment and therapeutic drug monitoring despite their potent bactericidal activity [26, 66].

The consequences are even more pronounced for agents whose toxicities substantially curtailed or displaced their therapeutic use. Colistin has re‐emerged as an important treatment for multidrug‐resistant Gram‐negative infections despite a narrow therapeutic window, in which increasing systemic exposure to improve bacterial killing also increases the risk of nephrotoxicity [12, 14, 55]. Its clinical use was further complicated historically by inconsistent dosing conventions, variable conversion of CMS to active colistin, and the absence of standardized pharmacokinetic guidance [52, 57]. International consensus recommendations introduced loading and exposure‐guided dosing strategies to improve efficacy while limiting toxicity, but the fundamental tension between antibacterial exposure and renal safety remains [58, 109]. Similar constraints extend beyond antibacterial therapy. Tartar emetic, an antimony‐based treatment historically used against leishmaniasis and schistosomiasis, was limited by severe systemic toxicity, particularly cardiotoxicity, contributing to its displacement by better tolerated therapies [15, 39]. Likewise, the systemic utility of membrane‐active agents such as gramicidin S has remained limited because concentrations required for antimicrobial activity can also disrupt mammalian membranes and cause substantial cytotoxicity [11, 16].

A narrow therapeutic index is therefore not simply a dosing inconvenience; it can determine whether otherwise potent antimicrobial activity can be exploited clinically. Conventional dose reduction may decrease toxicity at the cost of insufficient antimicrobial exposure, whereas dose escalation can improve pathogen killing while increasing off‐target injury. This trade‐off provides a particularly relevant rationale for advanced delivery systems. Rather than increasing total systemic exposure, nanocarriers can alter the distribution of an antimicrobial by concentrating it in infected tissues, controlling its release, or limiting exposure in toxicity‐prone organs [1, 18, 35]. The objective is therefore not necessarily to change the intrinsic toxicity of an antimicrobial molecule, but to increase the proportion of administered drug that reaches the desired site relative to healthy tissues. Such spatial and temporal control could effectively widen the usable therapeutic window of agents whose conventional formulations provide insufficient separation between antimicrobial efficacy and host toxicity.

### 2.5. Clinical, Regulatory, Commercial, and Historical Factors

The decline of older antimicrobial agents cannot be explained by pharmacological limitations alone. Many entered clinical practice before randomized clinical trials, standardized susceptibility breakpoints, and contemporary pharmacokinetic/pharmacodynamic (PK/PD) approaches became integral to antimicrobial development. Subsequent changes in regulatory requirements left some older agents with limited efficacy data or uncertain regulatory status despite continued microbiological activity [9, 116]. This legacy remains evident in inventories of “forgotten antibiotics,” which show substantial geographic variation in the licensing and availability of older antimicrobial agents, with particularly important access gaps in low‐ and middle‐income countries [22–24]. Fusidic acid illustrates this regulatory divergence: Despite decades of clinical use in several countries, it has not received approval for systemic use in the United States [86].

Commercial considerations have similarly influenced which antimicrobial agents remained in development or clinical use. Antibiotic development is characterized by high development costs, long timelines, and comparatively weak expected financial returns, reducing incentives to reinvest in older compounds once more attractive alternatives become available [5, 6]. Historical examples illustrate different manifestations of this problem. Nourseothricin was abandoned after early evidence of nephrotoxicity but revisited decades later when purification of individual streptothricin congeners revealed compounds with improved toxicity profiles and activity against multidrug‐resistant Gram‐negative pathogens [84]. Hygromycin A, by contrast, attracted limited development interest because of its narrow antibacterial spectrum before its selective activity against *Borrelia burgdorferi* renewed interest in its therapeutic potential [85]. Other agents disappeared for more conventional market reasons; spectinomycin, for example, was discontinued from the US market in 2005 due to limited availability and commercial demand rather than a loss of antimicrobial activity [117].

Economic barriers also persist for newly developed antimicrobials. The commercial failure surrounding plazomicin despite regulatory approval demonstrates that scientific and regulatory success does not necessarily translate into a sustainable antimicrobial market [6]. Moreover, revival of older agents introduces stewardship considerations that extend beyond formulation and efficacy. Colistin, for example, was extensively used in food‐producing animals, and such antimicrobial pressure contributed to the selection and dissemination of plasmid‐mediated colistin resistance, including *mcr-1*, with subsequent implications for both veterinary and human medicine [118, 119]. Reintroduction strategies must therefore consider not only whether an older antimicrobial can be made safer or more effective, but also how renewed use could influence resistance selection, accessibility, and responsible antimicrobial stewardship.

## 3. Nanotechnology as a Therapeutic Revival Catalyst

Nanotechnology provides a mechanistic framework for reconsidering antimicrobial agents whose therapeutic potential has historically been constrained by toxicity, instability, unfavorable pharmacokinetics, poor biodistribution, or narrow therapeutic windows. The central advantage is not necessarily the modification of the antimicrobial molecule itself, but the modification of the conditions under which the molecule reaches, persists within, and interacts with the biological target. Nanoenabled delivery systems can modify the physicochemical and pharmacological behavior of an incorporated agent by improving solubility, protecting labile compounds from premature degradation, regulating drug release, and altering systemic exposure and tissue distribution ([17, 18, 27, 35]; Figure 1). Importantly, these effects do not necessarily require modification of the antimicrobial molecule or its mechanism of action. Rather, engineering the carrier can change how much active agent reaches the intended site, how long it remains available, and the extent to which healthy tissues are exposed [1, 27]. This distinction is particularly relevant for older antimicrobial agents whose potent activity was retained but whose conventional delivery produced an unfavorable balance between efficacy and toxicity. Advances in nanocarrier design have further extended this concept beyond simple encapsulation to include spatial and temporal regulation of antimicrobial activity. Nanoparticle size, composition, surface properties, and route of administration can influence circulation, clearance, tissue accessibility, cellular uptake, and biodistribution [18, 27]. Responsive nanosystems can additionally exploit infection‐associated changes in pH, redox conditions, enzymes, and other microenvironmental signals to promote localized carrier disassembly or drug release [120]. These approaches increase antimicrobial exposure where needed while potentially limiting premature or off‐target exposure elsewhere. This principle is increasingly relevant across anti‐infective therapy, including antibacterial, antiviral, antifungal, and antiparasitic applications [39, 82, 121, 122]. Accordingly, the therapeutic‐revival potential of nanotechnology can be considered in terms of three interconnected functions: protecting both the antimicrobial payload and the host, controlling where and when antimicrobial activity occurs, and broadening the functional therapeutic window of historically constrained agents.

**Figure 1:**
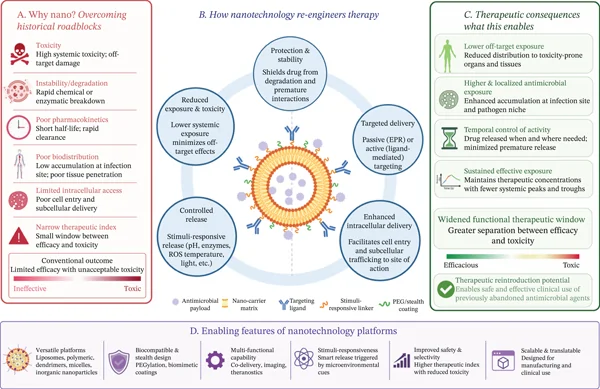
Nanotechnology as a therapeutic revival catalyst for historically constrained antimicrobial agents. Nanotechnology can address toxicity, instability, poor pharmacokinetics, inadequate biodistribution, limited intracellular access, and narrow therapeutic indices through payload protection, targeted delivery, controlled release, and altered biological exposure, thereby widening the functional therapeutic window and supporting therapeutic reintroduction.

### 3.1. Protecting the Antimicrobial While Limiting Host Exposure

A central advantage of nanoenabled delivery is its ability to physically or chemically isolate an antimicrobial payload from the surrounding biological environment until release is required. This is particularly relevant for agents whose therapeutic performance is compromised by degradation, rapid clearance, nonspecific interactions, or toxicity resulting from exposure of healthy tissues. Encapsulation within lipid, polymeric, or other nanoscale carriers can protect incorporated compounds from hydrolytic, enzymatic, and physicochemical degradation while modifying their circulation and release profiles [17, 18, 35]. The carrier therefore functions not simply as a vehicle but as an interface between the antimicrobial and the host, influencing the proportion of drug that remains encapsulated, its distribution among tissues, and the concentration of freely available agent during circulation [17, 27]. For highly reactive, membrane‐active, or otherwise cytotoxic compounds, reducing premature exposure may be as important as protecting the antimicrobial itself.

This principle is particularly relevant where the mechanism responsible for antimicrobial efficacy also contributes to host toxicity. Conventional administration exposes both infected and healthy tissues to the active compound, whereas encapsulation can modify biodistribution and reduce interactions with toxicity‐prone organs without altering the antimicrobial pharmacophore [1, 27]. Moreover, adequate systemic exposure does not necessarily translate into effective exposure within the pathogen′s niche, particularly for intracellular infections, where antimicrobials must traverse host‐cell membranes, navigate intracellular trafficking pathways, and retain activity within distinct subcellular environments [34]. Nanocarrier design can therefore serve two complementary purposes: limiting unnecessary host exposure while facilitating delivery of the antimicrobial to biological compartments that conventional formulations may inadequately access [34].

Amphotericin B provides an established example of this formulation‐dependent change in toxicity. Although its potent antifungal activity is retained, incorporation into lipid‐based and liposomal formulations alters tissue distribution and reduces nephrotoxicity compared with conventional amphotericin B deoxycholate, demonstrating how delivery engineering can improve the therapeutic profile of an intrinsically toxic antimicrobial [82]. Similar principles are being investigated beyond antifungal therapy. Liposomal encapsulation of tartar emetic, an antimonial historically constrained by systemic and cardiac toxicity, improved its pharmacokinetic profile and mitigated cardiotoxicity in an experimental murine model, illustrating the potential for formulation to reconsider compounds whose toxicity previously restricted therapeutic use [39].

Protection of the payload is equally important for compounds that are unstable or rapidly lost before reaching their intended site. Nanocarriers can provide a controlled physicochemical microenvironment that limits premature degradation or release, prolongs drug residence, and modifies systemic clearance [18, 35]. However, greater stability is not inherently advantageous if the carrier retains the antimicrobial too strongly or fails to release a pharmacologically effective fraction at the target site. Drug loading, carrier–payload interactions, release kinetics, carrier degradation, and biological conditions encountered after administration collectively determine whether encapsulation translates into improved antimicrobial exposure [17]. Thus, successful therapeutic revival requires a balance between protecting the agent during delivery and ensuring sufficient liberation of its active form at the site of infection.

Importantly, encapsulation should not be equated automatically with detoxification. Nanocarriers can redistribute rather than eliminate toxicity, and their physicochemical characteristics can promote accumulation in organs such as the liver and spleen or introduce carrier‐associated inflammatory, immunological, or cellular effects [27, 47]. The relevant measure of success is therefore not simply whether an older antimicrobial can be incorporated into a nanoparticle, but whether nanoformulations produces a meaningful improvement in the relationship between antimicrobial exposure and host injury. For historically constrained agents, this dual capacity, to preserve the active payload while reducing premature or off‐target host exposure, provides the first mechanistic basis through which nanotechnology can support therapeutic reintroduction.

### 3.2. Reprogramming Where and When Antimicrobial Activity Occurs

Beyond protecting antimicrobial payloads from degradation and limiting systemic toxicity, nanotechnology offers the possibility of controlling when and where antimicrobial activity is initiated, and under what biological conditions. Conventional formulations generally distribute active compounds based on their intrinsic physicochemical and pharmacokinetic properties, which can result in substantial exposure in healthy tissues while delivering only a fraction of the administered dose to infected sites. Nanoenabled systems can partially decouple administration from antimicrobial exposure by incorporating targeting, retention, and controlled‐release functions into the carrier itself [18, 27]. This distinction is particularly important for historically constrained agents whose antimicrobial activity is potent but difficult to confine spatially. Rather than attempting to fundamentally alter such agents, nanocarriers provide an opportunity to restrict their activity preferentially to the biological compartment in which it is required.

Spatial control can occur at multiple levels, from preferential accumulation within infected tissues to recognition of microbial surfaces and delivery into infected host cells. Nanoparticle size, charge, surface chemistry, and ligand functionalization can influence interactions with biological barriers, cellular uptake, and tissue retention, whereas pathogen‐ or host‐directed ligands can further enhance association with selected cellular or microbial targets [1, 27]. Importantly, the relevant therapeutic destination may extend beyond the infected organ itself. Intracellular pathogens occupy heterogeneous niches including phagosomes, lysosomes, cytosol, and other cellular compartments, where antimicrobial concentrations can differ substantially from plasma or whole‐tissue measurements [34]. Consequently, successful delivery may require progression across several spatial scales—organ → infected tissue → host cell → pathogen‐containing subcellular compartment. This has shifted attention from simply increasing cellular uptake toward controlling intracellular trafficking, subcellular localization, and local pharmacokinetics at the pathogen niche [34].

Temporal control adds a second dimension by maintaining the antimicrobial in a relatively protected state until conditions favor its release. Infection sites frequently differ from healthy tissues in acidity, oxidative and reductive conditions, enzyme activity, and inflammatory signaling, providing endogenous cues that can be incorporated into responsive carrier designs [120]. Dynamic covalent linkages, for example, can be engineered to remain comparatively stable during circulation but undergo cleavage or carrier disassembly in response to changes in pH or redox conditions, thereby promoting release within the infection‐associated microenvironment [120]. External stimuli, including light, ultrasound, magnetic fields, or heat, can provide additional control where the anatomical location and nature of the infection permit localized activation. Such strategies are particularly attractive for reactive or membrane‐active antimicrobial chemistries because therapeutic activity can, in theory, remain constrained until the carrier reaches the intended site, thereby reducing unnecessary interactions with host tissues.

The practical value of this approach is illustrated by increasingly sophisticated infection‐responsive delivery systems. Fu et al. [30], for example, developed dual‐responsive nanoparticles for pneumonia that combined inflammatory lung targeting with bacterial recognition and microenvironment‐responsive antibiotic delivery. In experimental models, the system enhanced rifampicin accumulation at infected lung tissue and improved antibacterial activity while reducing disruption of the intestinal microbiota compared with free antibiotic exposure [30]. Although such systems remain substantially more complex than conventional antimicrobial formulations, they demonstrate an important principle for therapeutic revival: The biological activity of a drug need not occur wherever the drug distributes after administration if its release can instead be coupled to infection‐associated location or conditions.

This principle is especially consequential for older antimicrobials whose historical limitations arose from indiscriminate reactivity rather than inadequate antimicrobial potency. Spatial targeting can increase the fraction of an administered dose reaching the desired compartment, whereas stimuli‐responsive release can reduce premature activation during circulation; together, these strategies may generate a greater difference between antimicrobial exposure at the infection site and exposure of healthy tissues [27, 120]. Nevertheless, targeting should not be interpreted as absolute specificity. Biological heterogeneity, variable expression of targeting determinants, nanoparticle clearance, differences between experimental infection models and human disease, and incomplete carrier activation can all reduce the selectivity achieved in vivo [27]. The translational objective is therefore not perfect localization, but a sufficiently favorable redistribution of antimicrobial exposure to improve efficacy relative to toxicity. When achieved, such spatiotemporal control provides the mechanistic bridge between nanoenabled delivery and the broader goal of expanding the functional therapeutic window of previously constrained antimicrobial agents.

### 3.3. Expanding the Functional Therapeutic Window

Ultimately, the therapeutic value of protecting an antimicrobial payload and controlling its spatial and temporal exposure depends on whether these modifications produce a meaningful separation between antimicrobial efficacy and host toxicity. This relationship can be considered a functional therapeutic window: The range of exposure over which sufficient antimicrobial activity can be achieved without unacceptable host injury. For toxicity‐limited antimicrobials, this window may be constrained when the exposure required for effective antimicrobial activity approaches that associated with host toxicity [1, 47]. Nanoenabled delivery provides an opportunity to modify this relationship because carrier properties can alter drug release, circulation, biodistribution, tissue exposure, and clearance without necessarily changing the molecular target or intrinsic antimicrobial mechanism of the incorporated agent [17, 18, 27, 35].

Widening the therapeutic window does not necessarily require increasing total systemic drug exposure. Instead, nanoformulation can be designed to increase antimicrobial exposure at the site of infection relative to exposure in toxicity‐prone tissues [1, 27]. Particle size, morphology, composition, surface chemistry, and route of administration influence nanoparticle circulation, biological interactions, tissue accessibility, cellular uptake, and clearance, thereby affecting where an associated therapeutic payload ultimately accumulates [18, 27]. Controlled and sustained‐release systems can additionally modify the rate and duration of payload availability, potentially maintain therapeutically relevant exposure while limiting premature release or high concentrations of free drug outside the intended site [17, 35]. This relationship becomes particularly important for intracellular infections because adequate plasma or tissue concentrations do not necessarily ensure adequate drug exposure within infected cells or the specific subcellular compartments occupied by pathogens [34]. Cellular entry, intracellular trafficking, subcellular localization, and the physicochemical conditions of pathogen‐containing compartments can therefore become determinants of antimicrobial exposure that are not captured by conventional plasma pharmacokinetics alone [34].

Evidence from toxicity‐limited anti‐infective agents demonstrates that such redistribution can translate into clinically or experimentally meaningful improvements in tolerability. Amphotericin B is an established example: lipid‐based and liposomal formulations alter its pharmacokinetic and tissue distribution and reduce nephrotoxicity compared with conventional amphotericin B deoxycholate, while retaining antifungal activity [82]. This formulation‐dependent reduction in toxicity demonstrates that the therapeutic utility of a potent antimicrobial can be improved by altering how the active compound interacts with and distributes within the host, rather than by replacing its antimicrobial pharmacophore [82]. A similar principle has been demonstrated experimentally with tartar emetic, an antimonial whose therapeutic use has historically been constrained by systemic toxicity. Liposomal encapsulation modified its pharmacokinetic profile and mitigated cardiotoxic effects in a murine model compared with the free compound [39]. Together, these examples support a central premise of therapeutic revival: An antimicrobial need not become intrinsically less toxic if delivery engineering can sufficiently redistribute the biological exposure responsible for both efficacy and toxicity [39, 82].

The therapeutic window may also be modified by controlling the magnitude and duration of antimicrobial release. Nanocarrier composition, drug–carrier interactions, degradation behavior, and surrounding biological conditions can determine the rate at which an encapsulated payload becomes pharmacologically available in vivo [17]. Sustained‐release approaches may prolong local drug availability and reduce the need for repeated administration in some applications, whereas stimuli‐responsive systems can restrict substantial payload release until predefined biological conditions are encountered [35, 120]. Nanoformulations can also support coencapsulation or coordinated delivery of multiple therapeutic components, creating opportunities to achieve complementary or synergistic activity while controlling their relative exposure [1]. However, reduced dosing, synergy, or prolonged release should not themselves be interpreted as evidence that the therapeutic index has improved. Demonstrating genuine therapeutic rescue requires evidence that the nanoformulations shift the exposure–response and exposure–toxicity relationships relative to the corresponding free or conventionally formulated antimicrobial.

Importantly, nanoparticle incorporation does not inherently widen the therapeutic window. Nanocarrier physicochemical properties also determine their distribution and clearance and can produce biological effects independent of the incorporated drug [27, 47]. Protein adsorption and interactions with immune and cellular systems can alter nanoparticle identity and biological behavior after administration, potentially changing both efficacy and toxicity [27, 47]. Moreover, substantial nanoparticle accumulation can occur within clearance organs, particularly components of the mononuclear phagocyte system such as the liver and spleen, meaning that reducing exposure in one tissue can be accompanied by increased carrier or payload exposure elsewhere [27]. Differences in nanoparticle biodistribution and biological interactions across experimental models further complicate extrapolation of favorable preclinical findings to humans [27, 47]. Consequently, successful therapeutic revival should not be defined simply by greater antimicrobial activity, successful nanoparticle loading, or reduced toxicity at a single dose. Rather, it requires evidence that the nanoformulations produce a reproducible and biologically meaningful improvement in the exposure–efficacy–toxicity relationship relative to the conventional agent.

## 4. Case Studies in Nanotechnology‐Driven Antimicrobial Reintroduction

The translational value of nanotechnology for historically constrained anti‐infective agents depends on more than improved antimicrobial activity. The central question is whether a nanoplatform addresses the specific chemical, pharmacological, or biological barrier that restricted the parent agent′s therapeutic use or enables an application that was previously infeasible. These barriers include uncontrolled chemical reactivity, organ‐specific toxicity, unfavorable pharmacokinetics, short reactive‐species lifetimes, poor membrane selectivity, limited spectrum, and formulation instability. Accordingly, the case studies assess whether nanoenabled formulations modify the properties underlying these limitations, rather than evaluating antimicrobial potency alone (Table 2).

**Table 2:** Nanotechnology‐enabled case studies relevant to previously abandoned antimicrobial agents.

Nanotechnology type	Nanoplatform/formulation	Antimicrobial drug(s) improved	Pathogen/model	Limitation targeted/potentiation	Reported outcome	Evidence level	Remaining translational limitation	References
2D nanomaterial (iodine allotrope)	H_2_O_2_‐responsive iodine nanosheet (iodinene)	Iodine	MRSA/*E. coli*; murine wound and pneumonia models	Uncontrolled, nonselective oxidative reactivity of free iodine causing tissue irritation	Weaker bactericide than povidone‐iodine at equal iodine concentration in vitro, but H_2_O_2_‐triggered allotropic transformation to HIO/I2 in situ gave efficacy in vivo that in vitro potency alone would not predict	In vivo (rodent)	Allotropic‐transition mechanism untested in larger animal or clinical models	[123]
Polysaccharide nanoparticle (photothermal)	Amylose‐inclusion iodine nanoparticles	Iodine (KI‐I2)	*E. coli*, MRSA; in vitro	Cytotoxicity of free iodide at bactericidal concentration	Bacterial survival reduced to under 2% within 5 min of near‐infrared laser exposure; lower endothelial cytotoxicity than free KI‐I2 solution	In vitro	No in vivo infection data reported	[124]
Metal‐organic‐framework composite	NIR‐triggered MOF coating (AuNR@SiO2@UiO‐66)	Iodine	Gram‐positive/negative bacteria; surface‐coating model	Uncontrolled release and poor spatial targeting of free iodine	Light‐triggered release combined with passive baseline release in a single antibacterial film	In vitro	Coating durability under real hospital‐surface conditions untested	[41]
MOF/photodynamic composite coating	MAO + ZIF‐8 NIR–triggered coating	Iodine	*S. aureus*‐infected rat tibial implant	Poor spatiotemporal targeting for implant‐associated infection	Lowest bacterial colony counts among all tested groups; improved osseointegration in vivo	In vivo (rodent, implant)	NIR penetration depth limits use to superficial or accessible implants	[125]
Peptide hydrogel	Self‐assembling peptide hydrogel (Nap‐FFGP, proline‐rich povidone mimic)	Iodine (PVP‐I analogue)	Murine MRSA‐induced endometritis	Iodophor‐carrier toxicity at effective dose	Antibacterial performance comparable with conventional iodophor with measurably higher cell viability in parallel cytotoxicity assays	In vivo (rodent)	Efficacy in nonmucosal, chronic wounds untested	[126]
Magnetic nanoparticle	Quaternized N‐chloramine‐coated magnetic (Fe3O4) NPs	N‐chloramine (class)	In vitro antibacterial and magnetic‐recovery testing	Formulation instability of the N–Cl bond; residual oxidant left behind after treatment	Superior antibacterial activity with minimal residual toxicity; magnetically recoverable from a treated surface or fluid after use	In vitro	No infection‐model or in vivo safety data	[127]
Nanotextured surface	Nanospike‐decorated polymer surface with quaternized N‐chloramine	N‐chloramine (class)	In vitro surface antibacterial testing	Formulation instability; single‐mechanism reliance limiting dose reduction	Enhanced antibacterial activity from combined mechanical membrane puncture and oxidative chloramine action, at lower active‐chlorine concentration than flat coatings	In vitro	Not tested against biofilm or in an infection model	[128]
Lipid nanocarrier (topical)	Lipid‐based nanocarrier	Clioquinol	Dermatophytes, *Candida* spp.; in vitro	SMON neurotoxicity restricting the drug to topical‐only use	Antifungal activity against dermatophyte and *Candida* strains retained in a formulation that never re‐enters systemic circulation	In vitro	Human dermatomycosis efficacy/safety data not yet available	[92]
Nanocrystal	Formulation‐optimized nanocrystals (method, drug concentration, and stabilizer varied)	Clioquinol	In vitro/physicochemical characterization	SMON neurotoxicity; poor aqueous solubility limiting topical delivery	Improved dissolution and topical‐delivery properties	In vitro (formulation)	No antifungal efficacy or in vivo data yet reported	[91]
Polymeric nanoparticle	PCL–pluronic composite nanoparticles	Chloramphenicol	MRSA‐infected murine burn wound	Dose‐independent aplastic anemia risk from systemic exposure	Localized, high‐concentration antibacterial activity at the wound with reduced systemic exposure	In vivo (rodent)	Systemic dosing regimen for nontopical indications unresolved	[69]
Silica/hydrogel carrier	Nanosilica/carbopol hydrogel carriers	Chloramphenicol	Reference bacterial strains; in vitro	Systemic toxicity limiting use beyond topical/ophthalmic application	Improved release profile and bacteriostatic activity	In vitro	Not tested against resistant or biofilm strains	[70]
Metal‐nanoparticle synergy conjugate	*γ*‐Cyclodextrin‐capped AgNPs; Mg‐doped ZnO NPs	Chloramphenicol	Various bacterial strains; in vitro	Targets antibacterial potency, not chloramphenicol′s own bone‐marrow toxicity	Enhanced antibacterial activity via molecular recognition within the cyclodextrin cavity, or via metal‐oxide synergy	In vitro	Does not address aplastic‐anemia risk; benefit is metal‐mediated, not halogen‐specific	[68]; [71]
Polysaccharide nanoparticle	Chitosan and cyclodextrin‐chitosan nanoparticles	Levofloxacin	Efflux pump‐resistant *E. coli*; ocular model	Efflux pump‐mediated resistance and poor ocular retention (not a historical toxicity failure)	Retained activity against resistant strains; roughly twofold potency gain against Gram‐negative ocular pathogens versus free drug	In vitro/ex vivo	Long‐term resistance‐suppression durability not established	[129]; [130]
Metal nanoparticle (topical)	AgNP topical cream	Silver	Human patients, culture‐positive infected wounds	Argyria; mammalian cytotoxicity at effective dose	Bacterial clearance and wound‐healing outcomes broadly comparable with mupirocin in an open‐label RCT	Human (RCT)	Long‐term safety/argyria risk with repeated use not established	[131]
Metal‐nanoparticle conjugate	AgNP–chlorhexidine conjugate	Silver + chlorhexidine	*S. aureus*, *E. coli*, *C. albicans*; in vitro	Combination toxicity poorly characterized	Increased antimicrobial synergy, but also measurably increased cytotoxicity relative to either agent alone	In vitro	Toxicity increase unresolved; not advanced to in vivo testing	[132]
Inhalable polysaccharide nanoparticle	HA/chitosan Ga(III) NPs, spray‐dried with mannitol	Gallium nitrate	Rat model; *P. aeruginosa*‐relevant	Narrow window of therapeutic lung concentration achievable by IV dosing before renal clearance	Significantly greater lung persistence of gallium alongside significantly lower plasma and urinary gallium than an equivalent IV dose	In vivo (rodent)	Human inhalation dosing/device translation not yet tested	[133]
Liquid‐metal nanostructure	Eutectic gallium‐indium (EGaIn) nanostructured films	Gallium (alloy)	*E. coli*, *S. aureus*; in vitro	Weak redox activity of ionic Ga(III) alone	Complete in vitro growth inhibition of both organisms via combined ROS generation and iron mimicry, a mechanism not shown by pure ionic Ga(III)	In vitro	No in vivo infection data	[134]
Bimetallic nanoparticle	Bismuth–silver bimetallic nanoparticles	Bismuth	*S. aureus*, *E. coli*; murine infected‐wound model	Weak intrinsic antibacterial potency of bulk bismuth	In vivo antibacterial efficacy without the fibroblast, endothelial, or epithelial cytotoxicity that constrains silver alone at comparable antibacterial concentrations	In vivo (rodent)	Mechanism not fully resolved; scale‐up untested	[135]
Liposome	Liposomal encapsulation (Lip‐TE)	Tartar emetic (antimony)	Murine pharmacokinetic/cardiac‐function model	Dose‐dependent cardiotoxicity (QT prolongation, conduction disturbance)	AUC0‐inf nearly tripled (~676 vs. 211 mg·L^−1^ min) and elimination half‐life more than doubled (462 vs. 178 min) versus free drug; cardiac antimony stayed below the detection limit; ejection fraction and QRS interval were preserved at 30 days, versus a significant, sustained decline with free drug	In vivo (rodent)	Repeat dosing triggers accelerated blood clearance, redirecting the drug toward greater hepatic accumulation	[39]; [95]
Liposome	Liposomal encapsulation (Lip‐TE)	Tartar emetic (antimony)	Leishmania‐infected mice	Systemic toxicity limits antileishmanial dosing	Reduced splenic and hepatic parasite burden relative to free drug	In vivo (rodent)	Human dosing and translation not yet established	[94]
Metal‐nanoparticle conjugate	Colistin‐conjugated selenium nanoparticles (Col‐SeNP)	Colistin	MDR *P. aeruginosa*, azole‐resistant *Candida*, MCF‐7; in vitro	Targets the mexY resistance‐efflux gene, not colistin′s own nephro‐/neurotoxicity	Downregulation of the mexY efflux‐pump gene associated with colistin resistance; retained activity against azole‐resistant *Candida*	In vitro	Nephro‐/neurotoxicity not addressed; no in vivo data	[136]
Metal‐nanoparticle synergy	AgNP + polymyxin B combination	Polymyxin B	*P. aeruginosa* biofilm (wound and cystic‐fibrosis‐relevant mucoid isolates)	Targets biofilm and resistance, not PMB′s own nephrotoxicity/neurotoxicity	Synergistic antibiofilm activity	In vitro	Nephro‐/neurotoxicity not addressed	[137]; [138]
Metal‐hybrid nanosphere	Colistin‐loaded polydopamine nanospheres decorated with AgNPs	Colistin	In vitro antibacterial/antibiofilm testing	Targets biofilm tolerance, not colistin′s own toxicity	Improved antibacterial and antibiofilm performance versus either component alone	In vitro	Nephrotoxicity/neurotoxicity not addressed; no in vivo data	[139]
Metal‐nanoparticle synergy	CuO, AgNP, and ZnO NP combinations; NIR‐activated AgNP	Fosfomycin	MDR, biofilm‐forming clinical isolates; in vitro	Targets bacterial resistance, not fosfomycin′s own salt form‐dependent oral bioavailability	Restored or potentiated activity against resistant, biofilm‐forming strains	In vitro	Does not address fosfomycin′s actual translational barrier (oral bioavailability)	[140]; [141]; [142]; [143]
Metal‐polymer synergy	AgNP + PLGA nanoparticle combination	Ketoconazole	*C. albicans*; in vitro	Targets antifungal potency, not hepatotoxicity/adrenal suppression	Enhanced antifungal synergy versus either agent alone	In vitro	Does not address ketoconazole′s actual translational barrier (hepatotoxicity)	[144]
Metal‐nanoparticle synergy	AgNP–vancomycin combination	Vancomycin	VRSA and related resistant strains	Rising resistance; nephrotoxicity at required doses	Synergistic activity against strains resistant to either agent alone	Mixed (in vitro/in vivo)	Optimal dose‐sparing ratio for clinical translation unresolved	[65]
NO‐donor nanoparticle	Silica/polymer/lipid NO‐releasing nanoparticles	Nitric oxide (class)	MRSA abscess, skin infection; murine	1–5 s biological half‐life; undeliverable by conventional pharmaceutics	Reduced lesion area and bacterial burden; preserved normal skin architecture	In vivo (rodent)	Manufacturing consistency and shelf‐life of NO donors not established	[43]
NO‐donor block‐copolymer assembly	Furoxan‐based NO donors coassembled with Pluronic F127	Nitric oxide (class)	MRSA; in vitro	Weak potency of free NO donors against biofilm	Fourfold higher antimicrobial activity versus the self‐assembled donor alone	In vitro	No in vivo biofilm data	[145, 146]
Janus nanoparticle (NO + photodynamic)	Ce6‐PDA/CaP‐GSNO Janus nanoparticles	Nitric oxide + photosensitizer	MRSA biofilm; in vivo	Poor biofilm penetration of NO donors alone	Biofilm‐core penetration achieved before NO release; combined NO/photodynamic‐therapy eradication of the biofilm	In vivo (rodent)	Light‐delivery depth limits use to accessible, superficial sites	[147]
Inhalable NO nanocarrier (conceptual)	Organ‐targeted/inhalable NO nanocarriers (proposed)	Nitric oxide (class)	*Mycobacterium tuberculosis* (proposed)	Systemic NO cytotoxicity precludes high‐dose exposure	Proposed dual mycobactericidal and macrophage‐clearance‐promoting mechanism	Preclinical/proposed	Human tuberculosis efficacy data not yet available	[42]
Metal‐sulfide fenton nanoplatform	CuS@CaO_2_@dextran H_2_O_2_‐self‐supplying nanoplatform	Reactive oxygen species (Fenton)	MRSA biofilm; murine wound	Insufficient endogenous H_2_O_2_; elevated glutathione quenches generated radicals	Substantial in vivo antibiofilm and wound‐healing efficacy under near‐infrared enhancement	In vivo (rodent)	Selectivity for infected versus healthy tissue not fully resolved	[148]
2D nanosheet (pH‐responsive)	pH‐responsive iron phosphosulfide (FePS3) nanosheets	Reactive oxygen species (fenton)	Biofilm/inflammation model	Nonselective host‐tissue oxidative damage from hydroxyl radicals	Selective ROS generation within acidic biofilm microenvironment; switches to ROS‐scavenging, anti‐inflammatory mode at neutral pH in healthy tissue	Mixed (in vitro/in vivo)	Long‐term biocompatibility of degradation products unresolved	[149]
Polymeric nanoparticle	Polymeric nanoparticles modulating intestinal permeability	Nitrofurantoin	Uropathogens; in vitro/formulation	Renal‐function‐dependent narrow safety margin	Improved formulation stability; recovered antivirulence activity distinguishable from direct bactericidal effect	In vitro	Renal‐impairment dosing safety not directly addressed	[150]
Mucus‐penetrating polymeric nanoparticle	Mucus‐permeable PMB‐HA/PLGA nanoparticles (nebulized)	Polymyxin B	Lung infection model	Nephrotoxicity/neurotoxicity; poor mucus penetration for nebulized delivery	Improved mucus penetration engineered specifically for nebulized administration	Mixed (in vitro/in vivo)	Aerosolisation/device compatibility at clinical scale untested	[62]
Polyelectrolyte complex/hydrogel	Hyaluronan/colistin polyelectrolyte complex; phenylboronic‐acid hydrogel	Colistin/polymyxin B	*P. aeruginosa*‐infected burn wound	Poor local retention; narrow therapeutic window	Extended antibiofilm activity well beyond free drug; wound‐responsive, triggered release	Mixed (in vitro/in vivo)	Human wound‐infection efficacy not yet tested	[151]; [152]
Coacervate nanoparticle	Coacervate complexation with polyanionic peptides (PEGylated, 8 nm)	Colistin	*A. baumannii* pneumonia; murine	Nephrotoxicity/neurotoxicity from systemic exposure	Improved survival in a pneumonia model at a matched dose versus free colistin	In vivo (rodent)	Manufacturing scale‐up of the coacervate process not established	[153]
Surface‐modified liposome	Chitosan surface‐modified nanoliposomes	Colistin	Colistin‐sensitive and ‐resistant clinical *P. aeruginosa*	Nephrotoxicity/neurotoxicity; emerging resistance	Improved activity against both colistin‐sensitive and colistin‐resistant isolates	In vitro	No in vivo toxicity/efficacy data	[154]
Nanoemulsion	Niclosamide‐colistin nanoemulsion and gel	Colistin	Colistin‐resistant Salmonella	Emerging colistin resistance	Restored efficacy via a dual‐agent lipid platform pairing colistin′s membrane action with an independent antibacterial contribution from niclosamide	Mixed (in vitro/in vivo)	Human/veterinary dosing translation untested	[126]
Nanoemulsion	Fisetin nanoemulsion coformulation	Polymyxin E (colistin)	Colistin‐resistant *S. Typhimurium*	Emerging colistin resistance	Enhanced efficacy through a plant‐flavonoid adjuvant mechanism	In vivo (rodent)	Flavonoid‐lipopeptide synergy mechanism not fully characterized	[59]
Clay‐based nanocomposite	Nanomontmorillonite‐loaded polymyxin B	Polymyxin B	Salmonella; ex vivo poultry model	Emerging resistance; veterinary stewardship concerns tied to the mcr‐1 reservoir	Long‐term antibacterial performance in an ex vivo poultry model	Ex vivo	Human/clinical translation not applicable (veterinary focus)	[155]
Antibody nanocombination	IgY antibody–polymyxin B nanocombination	Polymyxin B	Colistin‐resistant *S. typhimurium* (poultry isolate)	Emerging colistin resistance; food‐safety resistance reservoir	Combined antibody/antibiotic activity against a colistin‐resistant isolate	In vitro	Not yet tested in vivo or in human‐relevant models	[156]
Sonochemical nanosphere	Sonochemically processed vancomycin nanospheres	Vancomycin	*E. coli*, *P. aeruginosa* (Gram‐negative)	Complete exclusion from the Gram‐negative spectrum by outer‐membrane impermeability	10‐fold and 100‐fold sensitization of *E. coli* and *P. aeruginosa*, respectively, versus free vancomycin; electron microscopy confirmed physical outer‐membrane disruption, a mechanism independent of the conserved peptidoglycan target	In vitro	Not yet tested in vivo	[157]
Dendrimer conjugate	Multivalent dendrimer‐vancomycin conjugate	Vancomycin	Resistant Gram‐positive bacterial surface; in vitro	Reduced susceptibility in resistant strains	Restored or enhanced avidity‐driven activity against surfaces with reduced susceptibility	In vitro	Human pharmacokinetics of the dendrimer conjugate untested	[158]
Polymeric nanoparticle	Nanoparticle formulation of vancomycin	Vancomycin	VISA and MRSA strains	Narrow AUC/MIC therapeutic index	Retained activity against strains where free‐drug efficacy is marginal	In vitro	Nephrotoxicity reduction not directly measured	[159]
Gold nanoparticle‐peptide conjugate	AuNP conjugates with esculentin‐1a and LL‐37 peptides	Esculentin‐1a/LL‐37 (AMPs)	*P. aeruginosa*; wound‐healing model	Proteolytic instability; short circulating half‐life	Stabilized antipseudomonal and wound‐healing activity	Mixed (in vitro/in vivo)	Manufacturing cost and scale of the gold‐conjugate platform	[160]; [161]
Polymeric (PLGA) nanoparticle	PLGA and PEGylated‐PLGA nanoparticles	Colistin/SET‐M33 (AMPs)	*P. aeruginosa* lung‐infection model	Hemolysis and nephrotoxicity limit peptide dosing	Prolonged biofilm eradication versus free peptide; reduced toxicity with preserved antibacterial effect	In vivo (rodent)	Long‐term pulmonary biocompatibility of repeated dosing unresolved	[162]; [11]
Liposome	Liposomal encapsulation	Gramicidin S	Fibroblast monolayer/spheroid culture	Hemolytic and cytotoxic to mammalian cells since the 1940s	Protection factor exceeding 160‐fold against fibroblast cytotoxicity (80%–82% viability encapsulated versus 0.5% viability free), with membrane‐interacting activity preserved	In vitro	No antibacterial‐efficacy‐in‐infection‐model data in the same study	[163]
Polymer nanocontainer	Star‐like polyacrylamide nanocontainers with variable lipid composition	Gramicidin S	Model membrane systems; in vitro	Nonselective membranotropic action	Membrane selectivity shown to depend on carrier architecture, not on encapsulation alone	In vitro	Selectivity gains not yet tested in an infection model	[164]
Carbon‐based nanocarrier	Graphene oxide nanocarrier (GOGD)	Gramicidin	Bacterial strains; in vitro	Nonselective membrane toxicity	High antibacterial performance preserved	In vitro	Mammalian cytotoxicity of the graphene oxide carrier itself not fully resolved	[165]
Liposome (established)	Liposomal amphotericin B (AmBisome)—established clinical formulation	Amphotericin B	Systemic fungal infection; human	Nephrotoxicity via cholesterol‐membrane binding	First‐line clinical status achieved and sustained for decades	Human (clinical, established)	Cost and access remain barriers in low‐ and middle‐income countries	[81]
Topical nanoparticle	Amphotericin‐B‐releasing topical nanoparticle formulation	Amphotericin B	*Candida* spp.; burn wound (in vitro)	Nephrotoxicity restricts use to the IV/systemic route	72.4%–91.1% growth reduction against clinical *Candida* strains within 4 h	In vitro	Human burn‐wound efficacy trial not yet conducted	[166]

Abbreviations: AMP, antimicrobial peptide; AuNP, gold nanoparticle; HA, hyaluronic acid; MDR, multidrug‐resistant; MOF, metal–organic framework; MRSA, methicillin‐resistant *Staphylococcus aureus*; NIR, near‐infrared; NO, nitric oxide; PLGA, poly(lactic‐co‐glycolic acid); ROS, reactive oxygen species; VISA, vancomycin‐intermediate *Staphylococcus aureus*.

We purposively selected agents in Table 1 as representative examples of antimicrobials whose therapeutic use was abandoned or constrained by documented toxicity, chemical instability, unfavorable pharmacokinetics, or other limitations; this initial selection was not intended to be exhaustive. We identified case studies through targeted searches of PubMed and Google Scholar, last conducted in April 2026. Searches combined the names of antimicrobial agents listed in Table 1 with terms including “nanoparticle,” “nanocarrier,” “liposome,” “nanoformulation,” and “delivery,” supplemented by platform‐ or application‐specific terms where appropriate. No predefined publication‐date restriction was applied. These searches supported a narrative review rather than a systematic or exhaustive coverage. We selected studies primarily when they provided experimental evidence that a nanoplatform modified properties directly relevant to an agent′s established therapeutic limitations, including exposure, stability, localization, selectivity, or pharmacokinetic behavior. Section 4.2 additionally includes selected studies demonstrating enhanced activity against resistant strains without directly addressing the parent agent′s historical limitation. We explicitly discuss these as examples of nanoenabled potentiation, distinguishing improved antimicrobial activity from evidence supporting therapeutic reconsideration.

### 4.1. Halogen‐Based Agents

Among the agents considered here, iodine is a particularly suitable candidate for nanoenabled therapeutic re‐engineering because its broad antimicrobial activity is well established, whereas uncontrolled chemical reactivity, local irritation, and systemic toxicity have largely restricted its use to topical applications [114, 167]. Rather than increasing iodine′s intrinsic antimicrobial potency, contemporary nanoplatforms seek to control its chemical state, localization, and release. Two‐dimensional iodine nanosheets (iodinene), for example, exploit hydrogen peroxide within infected microenvironments to trigger allotropic transformation and release antimicrobial HIO and I_2_. Despite modest activity under baseline in vitro conditions, this H_2_O_2_‐responsive activation produced substantial antibacterial effects in murine wound‐infection and pneumonia models, demonstrating how infection‐associated chemistry can be used to spatially activate an otherwise highly reactive antimicrobial [123]. Amylose–iodine nanoparticles achieve control through sequestration rather than infection‐triggered transformation: Incorporation of polyiodide within the amylose helix reduced cytotoxicity toward human endothelial cells relative to free KI–I_2_ while retaining photothermal responsiveness and antibacterial activity against *Escherichia coli* and MRSA [124]. Externally triggered systems extend this concept further, with iodine‐containing metal–organic frameworks permitting low basal release followed by accelerated iodine liberation under near‐infrared irradiation [41, 125].

Other carrier systems similarly seek to regulate iodine availability rather than simply increase the administered concentration. Polymeric iodophors and povidone–iodine‐inspired nanosystems can stabilize iodine and modulate its release, extending the carrier principle underlying conventional iodophors into nanoscale formulations [38, 40, 168]. More recently, an iodine‐containing self‐assembling peptide hydrogel achieved antibacterial efficacy comparable with conventional iodophor in a murine MRSA‐induced endometritis model while demonstrating greater cell viability in parallel cytotoxicity experiments [126]. Although these studies remain predominantly preclinical, they support a common design principle: The therapeutic limitation of iodine may be addressed by controlling when, where and in what chemical form its antimicrobial reactivity is expressed, rather than attempting to eliminate that reactivity itself.

Clioquinol illustrates a different form of therapeutic revival. Its systemic use was discontinued following its association with subacute myelo‐optic neuropathy, creating a historical toxicity barrier to oral administration [90]. Nanoformulation does not necessarily overcome this liability by making systemic clioquinol intrinsically safer; instead, current approaches redirect the compound toward localized therapy. Polymeric nanocarrier and nanocrystal formulations have been investigated for the topical treatment of dermatomycosis, enabling high local antifungal exposure while minimizing the need for systemic administration [91, 92]. Clioquinol‐containing nanosystems have also been explored for cutaneous leishmaniasis, where localized delivery similarly offers a route to repurpose the compound without recreating the systemic exposure associated with its historical toxicity [93]. This distinction is important: Therapeutic revival need not reproduce the original route of administration; it may instead involve redesigning the conditions under which the agent can be used safely.

N‐chloramines represent a prospective, earlier stage extension of the same principle. Although these compounds possess broad antimicrobial activity and have been investigated as comparatively well‐tolerated topical anti‐infectives, their nanoenabled development remains limited [169]. Quaternized N‐chloramines have been immobilized on magnetically recoverable nanoparticles to combine antimicrobial activity with posttreatment removal, thereby reducing residual biocide exposure [127], whereas more recent nanospiked polymeric surfaces combine contact‐mediated mechanical disruption with oxidative N‐chloramine activity [128]. However, unlike the iodine systems, these approaches remain largely at the materials and in vitro stages and have yet to establish therapeutic rescue in infection models; hence, they represent prospective rather than demonstrated examples of nanoenabled reintroduction.

### 4.2. Metal‐Based Agents

The historical antimicrobial activity of metals does not imply that they are inherently resistance‐proof; microorganisms possess efflux, sequestration, and detoxification mechanisms that can reduce susceptibility to several metal ions [170]. Nanoengineering, therefore, does not eliminate the potential for resistance but can alter metal speciation, localization, release, and tissue exposure in ways that influence the balance between antimicrobial activity and host toxicity. Silver illustrates both the potential and the limitations of this strategy. Its antimicrobial activity involves multiple mechanisms, including membrane damage, disruption of protein function, oxidative stress, and interference with nucleic acids, whereas excessive or prolonged host exposure remains associated with cytotoxicity and systemic accumulation [98]. Topical AgNP formulations exploit high surface‐area‐to‐volume ratios and localized Ag^+^ release, and a randomized, open‐label clinical trial reported bacterial clearance and wound‐healing outcomes broadly comparable with those of topical mupirocin in patients with infected wounds [131]. However, nanoformulation does not inherently improve safety: conjugation of silver nanoparticles with chlorhexidine enhanced antimicrobial activity but also increased cytotoxicity relative to the individual components [132]. This example illustrates a limitation specific to combination nanoformulations—pairing agents for synergistic potency does not guarantee a favorable toxicity trade‐off.

Gallium provides a more direct example of nanoengineering addressing a defined delivery limitation. Ga(III) mimics Fe(III) and can enter microbial iron‐acquisition pathways but cannot undergo the same biologically required redox cycling, thereby disrupting iron‐dependent processes [171]. Although gallium has shown anti‐*Pseudomonas* activity, achieving and maintaining adequate concentrations at sites of pulmonary infection remains a delivery challenge. Costabile et al. [133] encapsulated Ga(III) within hyaluronic acid/chitosan nanoparticles and subsequently incorporated them into inhalable nanoembedded microparticles. Pulmonary administration increased gallium retention in rat lungs while reducing plasma and urinary exposure compared with an equivalent intravenous dose [133]. This is a clear example of pharmacokinetics‐directed therapeutic re‐engineering: Rather than increasing the intrinsic antimicrobial activity of gallium, nanoformulation redirected its pharmacokinetics toward the target organ while reducing systemic exposure. Other gallium nanomaterials may confer additional antimicrobial mechanisms; gallium‐based liquid‐metal systems, for example, have demonstrated antibacterial activity linked to oxidative stress and disruption of iron metabolism [134]. These systems are mechanistically interesting but represent the creation of new metal‐based antimicrobial properties more than rescue of the pharmacological limitations of conventional gallium therapy.

Tartar emetic provides an even clearer example of nanoenabled modification of a historically unfavorable therapeutic window. Its antiparasitic activity and dose‐limiting toxicity both arise from an antimony‐containing compound whose clinical use declined substantially as safer therapies became available [15]. Liposomal encapsulation markedly altered the pharmacokinetics of tartar emetic, increasing systemic exposure while reducing detectable antimony accumulation in cardiac tissue and attenuating cardiac functional abnormalities observed with the free drug in mice [39]. Importantly, liposomal tartar emetic also retained antileishmanial activity in experimental infection, indicating that reduced cardiac exposure was not achieved at the expense of therapeutic efficacy [94]. This combination, preservation of antimicrobial activity together with modification of the organ exposure responsible for dose‐limiting toxicity, makes tartar emetic one of the clearest preclinical examples of the therapeutic‐revival concept considered here. However, repeated administration introduces an important qualification. Accelerated blood clearance (ABC) of PEGylated liposomes has been reported to alter tartar‐emetic disposition and increase hepatic accumulation after subsequent doses [95]. Thus, nanoformulation may shift rather than eliminate exposure‐related liabilities, emphasizing the need to evaluate repeated‐dose pharmacokinetics and organ toxicity when assessing therapeutic rescue.

As anticipated in the methodological note at the start of this section, a different pattern dominates much of the metal–antimicrobial nanotechnology literature: Metal nanoparticles are frequently used as synergistic antimicrobial partners rather than as carriers designed to correct the historical limitation of the incorporated drug. Silver nanoparticles have potentiated polymyxin B activity against *P. aeruginosa*, while silver‐, copper oxide‐, and zinc oxide‐based systems have enhanced fosfomycin activity against resistant or biofilm‐forming bacteria [137, 138, 140–142]. Such combinations may lower the antimicrobial concentration required for activity or overcome resistance; however, these outcomes should not automatically be interpreted as therapeutic revival. Unless reduced effective doses translate into demonstrably lower host toxicity or improved pharmacokinetics, the original limitations of the partner drug, such as polymyxin‐associated toxicity or formulation and exposure constraints affecting fosfomycin, may remain unresolved. These studies therefore expose a central translational distinction: Nanoenabled potentiation demonstrates improved antimicrobial activity, whereas therapeutic revival requires evidence that the pharmacological liability responsible for historical restriction has itself been meaningfully corrected.

### 4.3. Reactive Oxygen and Nitrogen Species‐Based Agents

Reactive oxygen and nitrogen species represent a distinct challenge for antimicrobial development because the same chemical reactivity that enables broad microbial damage also limits their controllability as therapeutics [20, 21]. ROS and RNS can damage nucleic acids, proteins, membrane lipids, and essential metabolic machinery through oxidative and nitrosative reactions [20, 21]. However, their high reactivity, short biological lifetimes, and interactions with surrounding biomolecules make spatial and temporal control of antimicrobial exposure difficult [20, 43]. Nanotechnology therefore serves a somewhat different function here than for conventional small‐molecule antimicrobials: Nanoscale systems can provide localized reservoirs or reaction platforms from which reactive species are generated or released in a controlled manner, potentially restricting antimicrobial chemistry to the site at which it is required [42, 43].

Nitric oxide (NO) illustrates this principle particularly well. Its antimicrobial activity arises from multiple concentration‐ and environment‐dependent mechanisms, including DNA damage, protein modification, disruption of iron–sulfur clusters, and perturbation of microbial redox and metabolic processes [20, 42]. At lower concentrations, NO can also modulate biofilm behavior, whereas higher local NO exposure can contribute directly to antimicrobial activity [42]. Translating these properties into therapy is complicated by the short biological lifetime and rapid chemical transformation of NO, making sustained and localized exposure difficult to achieve with the freely generated molecule [42, 43]. Nanoparticle‐based NO donors address this limitation by storing NO or NO‐generating precursors and regulating their release through carrier composition, degradation, or environmental and externally applied stimuli [43].

Experimental studies provide evidence that such control can translate into antimicrobial activity rather than simply improved physicochemical stability. NO‐releasing nanosystems based on silica, polymeric, and lipid‐associated platforms have demonstrated antibacterial and antibiofilm effects in preclinical models, including MRSA‐associated infections [43]. More specifically, coassembly of a furoxan‐derived NO donor with Pluronic F127 nanoparticles increased antibacterial activity against MRSA compared with the corresponding self‐assembled NO donor, indicating that nanoscale organization itself can influence the biological performance of the NO‐delivery system [145, 146]. More elaborate platforms integrate NO delivery with complementary antimicrobial mechanisms. Amphiphilic Janus nanoparticles incorporating S‐nitrosoglutathione and a photosensitizer penetrated MRSA biofilms and combined NO release with photodynamically generated oxidative stress following light activation, producing greater biofilm eradication than the individual therapeutic components in experimental models [147]. Thus, NO nanotechnology has progressed from simply providing a reservoir for an unstable reactive species toward coordinating the location, timing, and mechanism of antimicrobial activity [43, 145–147].

A similar rationale applies to ROS‐generating nanoplatforms, where therapeutic performance depends not simply on generating oxidative damage but on restricting that damage sufficiently to preserve selectivity toward infected tissue [21, 43]. Contemporary systems therefore exploit infection‐associated microenvironmental conditions or externally applied energy to regulate ROS generation. Zhao et al. [148], for example, developed a copper sulfide/calcium peroxide‐based nanosystem that supplied H_2_O_2_ while simultaneously depleting glutathione, thereby reducing an important antioxidant defense against the generated oxidative species. Under acidic conditions associated with the biofilm microenvironment, this system amplified oxidative antibacterial activity, which was further enhanced by near‐infrared irradiation, and demonstrated therapeutic activity in an experimental MRSA biofilm infection model [148]. In a complementary approach, an iron phosphosulfide nanoagent exhibited pH‐dependent behavior, promoting ROS‐mediated antibacterial activity under acidic biofilm‐associated conditions while displaying ROS‐scavenging activity under near‐neutral conditions intended to approximate surrounding noninfected tissue [149]. These systems address a fundamental limitation of reactive‐species therapy: ROS that damage microbial macromolecules can also injure host biomolecules when their generation is insufficiently localized [21]. Coupling ROS production to infection‐associated conditions or externally controlled activation therefore provides a strategy for converting indiscriminate oxidative chemistry into spatially regulated antimicrobial activity [148, 149].

### 4.4. Membrane‐Active Agents

Membrane‐active anti‐infectives provide a particularly direct test of nanoenabled therapeutic re‐engineering because the physicochemical interactions responsible for antimicrobial activity can also contribute to host toxicity. Polymyxins, antifungal polyenes, gramicidins, and many antimicrobial peptides interact with microbial membrane components or perturb membrane organization, but insufficient discrimination between microbial and mammalian membranes can narrow their therapeutic window [16, 56, 80, 172]. For these agents, nanoformulation cannot eliminate membrane activity without compromising the mechanism being preserved. Its more relevant function is therefore to control where when, and at what concentration the membrane‐active compound encounters biological membranes, thereby altering the relationship between antimicrobial exposure and host toxicity [11, 56].

Polymyxins illustrate this challenge particularly well. Colistin and polymyxin B were largely displaced from systemic use following the introduction of better tolerated antibiotics because nephrotoxicity and neurotoxicity constrained their clinical application, but subsequently re‐emerged as important therapies against multidrug‐resistant Gram‐negative infections [12, 14, 52]. Their contemporary use remains complicated by dose‐limiting toxicity, complex pharmacokinetics, and a narrow therapeutic window [55, 57, 58, 109]. Nanoenabled approaches have therefore sought to alter drug disposition and concentrate exposure at infected sites rather than modify the intrinsic membrane‐active mechanism. Wu et al. [61, 62] developed a mucus‐permeable hyaluronic acid‐coated PLGA nanoparticle for nebulized polymyxin B delivery that achieved more than twofold greater mucus penetration than free polymyxin B in vitro, improved delivery to infected alveolar cells following nebulization in vivo, reduced drug‐associated toxicity, and improved therapeutic efficacy in a murine lung‐infection model [62]. Colistin nanoparticles formed through coacervate complexation with polyanionic peptides likewise reduced colistin‐associated toxicity while retaining antibacterial activity and improving therapeutic outcomes in a murine *Acinetobacter baumannii* pneumonia model [153]. Localized polymeric systems have also been developed for polymyxin delivery in wounds and biofilms, including phenylboronic‐acid–functionalized hydrogels for controlled polymyxin B release and hyaluronan/colistin polyelectrolyte complexes [151, 152].

Amphotericin B provides the strongest clinical precedent for this principle. Its antifungal activity depends principally on interactions with ergosterol‐containing fungal membranes, whereas interactions with mammalian membranes and the distribution of conventional formulations contribute to its dose‐limiting toxicity [80, 81]. Liposomal amphotericin B alters the pharmacokinetic and tissue‐distribution profile of the parent compound and achieves higher plasma and tissue concentrations with lower toxicity than amphotericin B deoxycholate, thereby improving its therapeutic index [81, 82]. Unlike most nanoenabled antimicrobial strategies discussed here, this principle has progressed beyond experimental proof of concept: Liposomal amphotericin B has accumulated decades of clinical use and is incorporated into international treatment guidelines for multiple systemic fungal infections [81]. Localized nanoparticle formulations extend the same concept; amphotericin B‐releasing nanoparticles have demonstrated antifungal activity in experimental *Candida*‐infected burn wounds, providing an alternative means of concentrating drug exposure at the site of infection [166]. Amphotericin B therefore provides perhaps the clearest clinical demonstration that delivery engineering can preserve an established antimicrobial mechanism while changing the exposure–toxicity relationship sufficiently to transform therapeutic usability.

Antimicrobial peptides represent an earlier stage of the same translational problem. Despite potent antimicrobial activity, clinical development of many peptides has been constrained by proteolytic degradation, short biological half‐life, unfavorable pharmacokinetics, and cytotoxicity at therapeutically relevant concentrations [11, 113]. Nanoparticle conjugation or encapsulation can protect peptide cargo from degradation, modify its interaction with host membranes, and concentrate exposure at infected tissues [160, 162]. Gold nanoparticles conjugated with esculentin‐1a(1–21)NH_2_, for example, enhanced antipseudomonal performance while reducing toxicity toward human keratinocytes compared with the free peptide under the conditions examined [160]. Engineered nanoparticles have similarly been investigated for local pulmonary delivery of cationic antimicrobial peptides against *P. aeruginosa*, where carrier‐mediated delivery was designed to protect peptide activity and overcome extracellular barriers encountered in the infected lung [162]. Gold nanoparticle conjugation has also been explored for LL‐37–based antimicrobial systems [161]. However, these approaches remain predominantly preclinical, and enhanced antimicrobial potency alone cannot demonstrate that the stability, pharmacokinetic, or host‐toxicity constraints that limit peptide translation have been overcome [11, 113].

Gramicidin S provides a direct experimental test of whether a nanoformulation can mitigate the toxicity of an intrinsically membrane‐active antimicrobial. The cyclic peptide exhibits potent antimicrobial activity through membrane interactions but also substantial mammalian cell toxicity, which has largely restricted its therapeutic application to localized use [16, 172]. Recent liposomal formulations provide direct evidence that carrier sequestration can alter this toxicity relationship. At 75 *μ*g/mL, free gramicidin S reduced L929 fibroblast viability to approximately 0.5%, whereas liposomal formulations preserved approximately 80%–82% viability at the same nominal concentration, corresponding to protection factors exceeding 160‐fold [163]. The protective effect was also observed in three‐dimensional fibroblast spheroids and varied according to liposomal lipid composition, demonstrating that the biological effect depends on carrier architecture rather than encapsulation alone [163]. Other nanocarrier studies similarly indicate that the membrane interactions of gramicidin S can be modulated by carrier composition; star‐like polyacrylamide nanocontainers containing different lipid environments altered its membranotropic behavior [164]. Although these findings remain preclinical and require confirmation in infection models, they provide clear proof of principle that nanoengineering can substantially reduce host‐cell exposure to an intrinsically membrane‐active compound without altering the antimicrobial pharmacophore.

Vancomycin represents a related but mechanistically distinct example and should therefore be considered separately from the membrane‐disrupting agents above. Its principal antibacterial mechanism is inhibition of peptidoglycan synthesis rather than direct membrane disruption, while its lack of conventional activity against Gram‐negative organisms is strongly influenced by the Gram‐negative outer membrane′s exclusion of the bulky glycopeptide from its target. Nanoengineering has been investigated as a means of circumventing this permeability barrier: Nanotransformation of vancomycin increased its activity against *E. coli* and *P. aeruginosa*, accompanied by alterations of the bacterial envelope [157]. Dendrimer‐based multivalent vancomycin and vancomycin‐loaded nanoparticles have separately been investigated as strategies to enhance bacterial targeting or activity against resistant Gram‐positive organisms [158, 159]. These approaches are better interpreted as nanoenabled spectrum or potency expansion than as rescue of a membrane‐active agent, but they illustrate how nanotechnology can alter biological access to an established antimicrobial target.

Across the 51 nanoplatform case studies summarized in Table 2, evidence maturity varies substantially: 26 have been evaluated only in vitro or in physicochemical/formulation studies, a further two in vitro/ex vivo or ex vivo alone, 14 in vivo rodent studies, and six report mixed in vitro/in vivo evidence. Only two platforms have reached human evaluation—an open‐label randomized trial of topical silver nanoparticles [131] and the long‐established clinical use of liposomal amphotericin B [81]—whereas one, an inhalable NO carrier proposed for tuberculosis, remains at the conceptual stage [42]. Except for liposomal amphotericin B, nanoenabled therapeutic revival remains predominantly preclinical.

## 5. Barriers to Clinical Translation, Regulation, and Implementation

The ability of nanotechnology to overcome a historical limitation of an antimicrobial does not necessarily translate into a clinically superior therapy. As illustrated by previous case studies (Table 2), nanoformulations can reduce off‐target exposure, alter pharmacokinetics and biodistribution, stabilize or control the release of reactive antimicrobial payloads, and increase drug exposure at sites of infection ([1, 18, 47, 49]; Figure 2). However, these advantages can come with formulation‐specific liabilities, including carrier toxicity, immune interactions, altered biodistribution, manufacturing variability, and difficulty predicting human pharmacokinetics ([44, 47, 49]; Figure 2). Therapeutic revival therefore requires more than demonstrating improved antimicrobial activity after nanoformulation. The relevant benchmark is whether the formulation reproducibly improves the parent agent′s exposure–efficacy–toxicity relationship while remaining sufficiently safe, manufacturable, regulatable, and economically viable for clinical implementation [44, 46, 49]. This distinction is particularly important for previously abandoned or therapeutically constrained antimicrobials, because nanoengineering may resolve the limitation that originally restricted their use while simultaneously introducing new barriers to translation (Figure 2).

**Figure 2:**
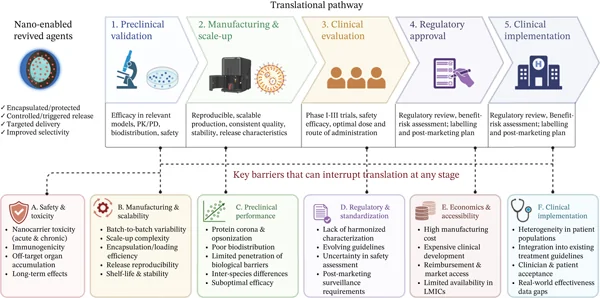
Translational pathway and barriers to clinical implementation of nanoenabled revived antimicrobial agents. Nanoenabled antimicrobial candidates must progress from preclinical validation through scalable manufacturing, clinical evaluation, regulatory approval, and ultimately clinical implementation.

### 5.1. Safety, Biocompatibility, and Long‐Term Biological Interactions

Reducing the toxicity of a historically constrained antimicrobial does not necessarily make the resulting nanoformulation intrinsically safe. Although nanoengineering can reduce off‐target exposure or alter the biodistribution of toxic payloads, nanoparticle composition, size, surface chemistry, and degradation can introduce additional interactions with plasma proteins, immune cells, and clearance organs [44, 47, 49]. Cardiovascular nanomedicine discusses a similar balance between localized delivery and systemic safety, where plaque‐targeted formulations aim to concentrate therapeutic payloads within diseased tissue while limiting off‐target exposure [173, 174]. This provides a useful comparison for antimicrobial delivery, although safety benefits must be established independently for each formulation and infection setting. For inorganic nanomaterials, ion release, oxidative stress, inflammatory responses, and tissue persistence may further contribute to toxicity, particularly during repeated administration [47]. Repeated exposure can also alter nanoparticle disposition through immune recognition. Polyethylene glycol (PEG), commonly used to prolong circulation, can interact with pre‐existing or treatment‐induced anti‐PEG antibodies and contribute to hypersensitivity or ABC, potentially changing drug exposure during repeated dosing [37, 49]. This concern is directly illustrated by PEGylated liposomal tartar emetic: Although encapsulation improved pharmacokinetics and reduced cardiac exposure, repeated administration promoted accelerated clearance and greater hepatic accumulation, showing that mitigation of one organ‐specific toxicity may redistribute exposure elsewhere [39, 95].

For abandoned or therapeutically constrained antimicrobials, carrier‐associated risks coexist with the intrinsic liabilities of the parent agent. Encapsulation controls rather than eliminates biological reactivity; premature leakage, carrier degradation, or uncontrolled release can therefore re‐expose host tissues to the toxicity that originally constrained therapeutic use [17]. Moreover, nanoformulation can generate effects not predictable from either component alone. The chlorhexidine–silver nanoparticle conjugation discussed in Section 4.2 illustrates this concern: Enhanced antibacterial activity was accompanied by increased cytotoxicity, demonstrating that nanoscale combination can amplify toxicity as well as efficacy [132]. Safety assessment should therefore distinguish between toxicity retained from the parent antimicrobial, toxicity introduced by the nanocarrier, and emergent toxicity arising from their interaction. Demonstrating reduced toxicity at a single dose or in one organ is insufficient to establish therapeutic rescue; the safety advantage must persist across relevant exposure and repeated‐dosing conditions [44, 47, 49].

### 5.2. Manufacturing and Scalability

Translation of nanoenabled antimicrobials from laboratory formulations to reproducible industrial production remains a major challenge [48, 51]. Therapeutic performance depends on critical formulation attributes including particle size and distribution, drug loading, encapsulation efficiency, surface properties, structural stability, and release kinetics, all of which can be altered by manufacturing conditions [44, 49, 50]. Consequently, process variability can directly translate into changes in biodistribution, drug exposure, efficacy, and toxicity, making batch consistency particularly important when a nanoformulation is intended to widen the narrow therapeutic window of a historically constrained antimicrobial [44, 47, 51]. Scale‐up further introduces challenges that may not be apparent during small‐batch laboratory production, including maintaining uniform particle characteristics, drug loading, and release behavior across larger manufacturing volumes [48, 51]. These requirements necessitate robust process control, quality‐by‐design approaches, and analytical methods that link critical quality attributes to therapeutic performance [50, 51, 175]. The absence of fully harmonized characterization and manufacturing standards further complicates reproducibility and regulatory evaluation [44, 46]. Thus, for therapeutic revival, manufacturing reproducibility is not merely a production requirement; it is necessary to ensure that the pharmacological advantage conferred by nanoengineering is consistent from batch to batch.

### 5.3. Pharmacokinetic Complexity and Clinical Performance

Although nanoengineering is often intended to improve pharmacokinetics and biodistribution, nanoparticle behavior is governed by both the encapsulated antimicrobial and carrier‐specific properties, including particle size, surface chemistry, protein adsorption, interactions with biological barriers, and immune clearance [18, 47, 49]. Consequently, favorable pharmacokinetic profiles observed in preclinical models may not translate directly to humans, where differences in physiology, immune responses, disease state, and nanoparticle disposition can alter exposure and therapeutic performance [18, 47]. Cardiac gene therapy faces related delivery challenges, where tissue tropism, immune responses, and differences between preclinical models and human physiology can constrain clinical translation [173, 174]. Although the therapeutic context differs, this comparison reinforces the need to establish delivery specificity and biological performance in models relevant to the intended clinical application. Infection‐site heterogeneity adds another layer of complexity. Vascularization, tissue architecture, inflammation, biofilm formation, local pH, and intracellular localization can influence nanoparticle penetration, payload release, and the concentration of pharmacologically active drug reaching the pathogen [17, 176]. For intracellular infections, plasma or whole‐tissue drug concentrations may poorly reflect exposure within infected cells and pathogen‐containing compartments, making subcellular drug delivery a key determinant of efficacy [34]. Thus, conventional measurements of total drug concentration may fail to distinguish among encapsulated, released, and pharmacologically available antimicrobial fractions. For nanoenabled therapeutic revival, pharmacokinetic success should be defined by whether reformulation reproducibly increases active drug exposure at the relevant pathogen niche while limiting exposure at toxicity‐prone sites. This requires comparative PK/PD studies of the nanoformulation and free parent agent, including clinically relevant dosing schedules and appropriate infection models [18, 49]. Without such exposure–response evidence, improved efficacy in in vitro or animal studies cannot establish that the historical pharmacokinetic limitation of the parent antimicrobial has been overcome.

### 5.4. Regulatory Challenges and Standardization

Regulatory evaluation of nanotechnology‐enabled therapeutics requires product‐specific assessment of how nanoscale properties influence quality, safety, and efficacy. The FDA′s 2022 guidance, “Drug Products, Including Biological Products, that Contain Nanomaterials,” explicitly recognizes that nanomaterials may confer attributes that warrant examination. Evaluation therefore extends beyond the active ingredient to particle characteristics, manufacturing controls, stability, release behavior, and biological disposition [177]. These considerations align with broader nanomedicine frameworks that emphasize integrating formulation characterization, reproducible manufacturing, biological evaluation, and regulatory planning throughout development [178]. For therapeutically constrained antimicrobials, historical clinical and pharmacological data remain informative but cannot automatically establish a nanoformulation′s safety or efficacy. Carrier‐dependent changes in biodistribution, drug release, and immune interactions can modify both therapeutic exposure and toxicity, so assessment should focus on the complete formulation rather than the parent agent alone [179]. The extent of additional physicochemical characterization, nonclinical testing, and clinical evaluation should therefore reflect the formulation′s properties, intended use, and differences from the established product [177]. Limited international harmonization of terminology, characterization methods, reference materials, and reporting standards further complicates comparisons across formulations [46, 49]. For nanoenabled antimicrobial revival, early regulatory engagement and reproducible evidence linking formulation quality to biological performance are therefore important for translating experimental improvements into clinically viable products [177, 178].

### 5.5. Economic Feasibility and Translational Implementation

Scientific feasibility does not guarantee clinical adoption. Re‐engineering an established antimicrobial may avoid some costs associated with discovering a new chemical entity, but nanoformulation introduces expenses for formulation development, specialized manufacturing, quality control, regulatory evaluation, and clinical validation [47, 49, 180]. Commercial viability and manufacturing feasibility should therefore inform development from an early stage, consistent with the DELIVER framework for nanomedicine translation [178]. These additional demands may increase the cost of an otherwise inexpensive antimicrobial, potentially reducing the economic advantage of repurposing. This challenge is particularly relevant to anti‐infective development, where weak commercial incentives and poor expected returns have contributed to market failures despite clinical need [5, 6]. For nanoenabled revival, the magnitude and clinical relevance of therapeutic benefit must justify the additional development and manufacturing costs. Improved antimicrobial activity alone does not establish this value; evaluation should also consider safety, dosing requirements, treatment delivery, and the intended clinical setting. Affordability and access matter especially in low‐ and middle‐income settings, where AMR imposes a substantial burden and resources for advanced therapies may be limited [1, 32, 136]. Successful implementation therefore requires formulations that deliver meaningful clinical benefit, can be manufactured reproducibly at scale, and are affordable, accessible, and compatible with antimicrobial stewardship.

## 6. Future Perspectives and Conclusion

Nanotechnology provides an opportunity to reconsider antimicrobial agents whose clinical utility was historically constrained by toxicity, instability, poor biodistribution, unfavorable pharmacokinetics, or insufficient selectivity. Nanoformulation can address these limitations through controlled release, altered tissue distribution, protection of unstable payloads, and localized antimicrobial activity [18, 47, 181]. However, the maturity of this concept varies considerably across agents, with many nanoenabled antimicrobial strategies remaining at the preclinical stage and facing substantial barriers to clinical translation [47, 181]. Future development should therefore be limitation‐driven rather than platform‐driven. The historical reason for therapeutic failure should define both the engineering objective and the experimental endpoint used to demonstrate rescue. For an agent limited by organ toxicity, increased antimicrobial potency alone is insufficient without evidence that the relevant toxicity or exposure has been reduced; similarly, compounds constrained by instability, poor tissue penetration, or intracellular accessibility require direct demonstration that nanoformulation corrects these deficiencies [18, 34, 49]. For intracellular pathogens in particular, successful delivery requires consideration of the cellular and subcellular compartments in which therapeutically relevant exposure must be achieved [34].

Accordingly, improved in vitro activity should not, by itself, be considered evidence of therapeutic revival. A stronger translational benchmark is a reproducible improvement in the exposure–efficacy–toxicity relationship relative to the parent agent, supported by appropriate pharmacokinetic, biodistribution, efficacy, and safety evidence. Translation must also account early for manufacturing reproducibility, scalability, regulatory requirements, commercial viability, accessibility, and antimicrobial stewardship rather than treating these considerations as downstream problems [46, 47, 181]. These requirements are consistent with recent calls for the development of nanoantimicrobials to integrate scientific, manufacturing, regulatory, economic, and stewardship considerations early in the translational pathway [181]. Ultimately, nanotechnology should not be viewed as a universal solution for failed antimicrobials, nor should every nanoformulation of an older compound be regarded as therapeutic revival. Its greatest value lies in enabling the rational re‐engineering of antimicrobial agents that retain biological utility but were constrained by limitations that contemporary delivery technologies may now be able to address. The future anti‐infective pipeline may therefore be expanded not only through discovery of new chemical entities, but also by revisiting established antimicrobial agents with technologies specifically designed to overcome the barriers that previously restricted their therapeutic use.

**Nomenclature**
ABC: accelerated blood clearance
AgNPs: silver nanoparticles
AMPs: antimicrobial peptides
AMR: antimicrobial resistance
AUC: area under the concentration–time curve
AUC/MIC: area under the concentration–time curve to minimum inhibitory concentration ratio
AuNPs: gold nanoparticles
CMS: colistin methanesulfonate
EMA: European Medicines Agency
FDA: US Food and Drug Administration
MDR: multidrug‐resistant
MIC: minimum inhibitory concentration
MOF: metal–organic framework
MRSA: methicillin‐resistant *Staphylococcus aureus*
NIR: near‐infrared
NO: nitric oxide
PCL: poly(*ε*‐caprolactone)
PEG: polyethylene glycol
PK: pharmacokinetics
PK/PD: pharmacokinetics/pharmacodynamics
PLGA: poly(lactic‐co‐glycolic acid)
PMB: polymyxin B
RCT: randomized controlled trial
RNS: reactive nitrogen species
ROS: reactive oxygen species
VISA: vancomycin‐intermediate *Staphylococcus aureus*
VRSA: vancomycin‐resistant *Staphylococcus aureus*

## Author Contributions

Babatunde Ibrahim Olowu: conceptualization, visualization, writing—original draft, writing—review and editing, writing—final draft. Olalekan Toheeb Okewale: visualization, writing—original draft, writing—review and editing, writing—final draft. Abdulhakeem Opeyemi Azeez: writing—original draft, writing—review and editing, writing—final draft. Shammah Oluwaseyi Adeyemi: visualization, writing—original draft.

## Funding

No funding was received for this manuscript.

## Disclosure

All authors reviewed and accepted the final draft of the manuscript.

## Ethics Statement

The authors have nothing to report.

## Consent

The authors have nothing to report.

## Conflicts of Interest

The authors declare no conflicts of interest.

## Data Availability

Data sharing is not applicable to this article, as no datasets were generated or analyzed during the current study.
